# Advancements in the impact of human microbiota and probiotics on leukemia

**DOI:** 10.3389/fmicb.2024.1423838

**Published:** 2024-07-03

**Authors:** Yi Zhang, Xiaotong Zhao, Jingxian Zhang, Yaodong Zhang, Yongjun Wei

**Affiliations:** Henan Key Laboratory of Children's Genetics and Metabolic Diseases, School of Pharmaceutical Sciences, Children’s Hospital Affiliated to Zhengzhou University, Henan Children’s Hospital Zhengzhou Children’s Hospital, Zhengzhou University, Zhengzhou, China

**Keywords:** gut microbiota, leukemia, probiotics, zebrafish models, leukemia treatment

## Abstract

The human gut microbiota is a complex ecosystem that plays a crucial role in promoting the interaction between the body and its environment. It has been increasingly recognized that the gut microbiota has diverse physiological functions. Recent studies have shown a close association between the gut microbiota and the development of certain tumors, including leukemia. Leukemia is a malignant clonal disease characterized by the uncontrolled growth of one or more types of blood cells, which is the most common cancer in children. The imbalance of gut microbiota is linked to the pathological mechanisms of leukemia. Probiotics, which are beneficial microorganisms that help maintain the balance of the host microbiome, play a role in regulating gut microbiota. Probiotics have the potential to assist in the treatment of leukemia and improve the clinical prognosis of leukemia patients. This study reviews the relationship between gut microbiota, probiotics, and the progression of leukemia based on current research. In addition, utilizing zebrafish leukemia models in future studies might reveal the specific mechanisms of their interactions, thereby providing new insights into the clinical treatment of leukemia. In conclusion, further investigation is still needed to fully understand the accurate role of microbes in leukemia.

## 1 Introduction

Hematopoiesis is a tightly regulated process that involves the differentiation and maturation of stem cells into various types of blood cells, including red blood cells, megakaryocytes, and immune cells of different lineages (Ribatti and d’Amati, 2023). However, genetic errors, such as cross-chromosome transfer, chromosome deletion, point mutation, and epigenetic changes, can disrupt the normal maturation of stem cells during hematopoiesis. This can lead to uncontrolled proliferation of immature leukemic cells, resulting in leukemia (Lin and Aplan, 2004). Therefore, leukemia is characterized by the clonal proliferation of the leukemic cells in the bone marrow. It often leads to an increased number of affected cells in the blood circulation, and in some cases, abnormal proliferation of lymphoid tissue may occur in lymphoid malignancies (Bispo et al., 2020). Leukemia is a type of Cancer that affects hematopoietic stem cells, which disrupts the production of normal blood cells. Leukemia can be divided into four main subgroups of acute myeloid leukemia (AML), acute lymphoblastic leukemia (ALL), chronic myeloid leukemia (CML), and chronic lymphoblastic leukemia (CLL) (Wang et al., 2019).

**Figure 1:**
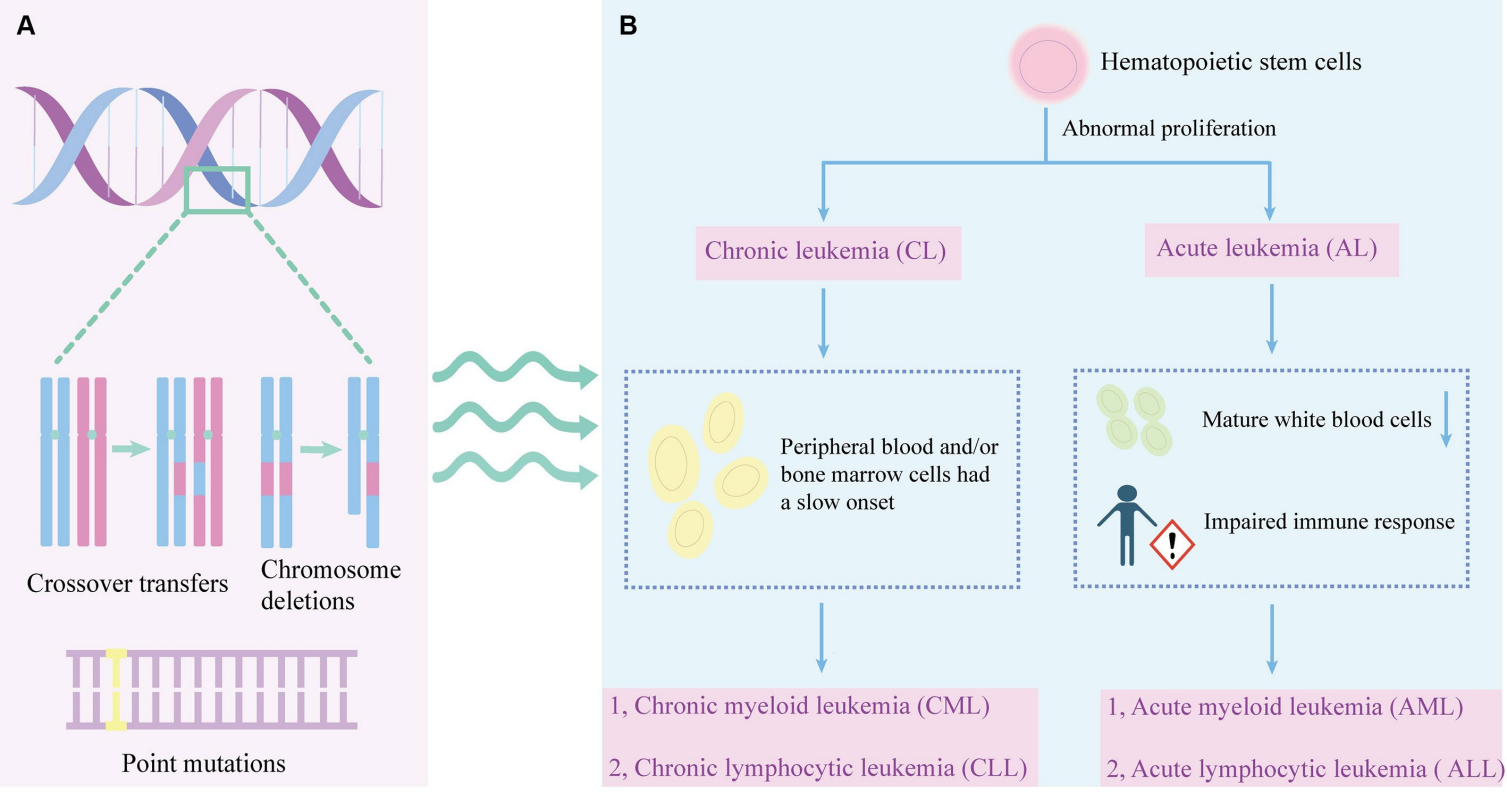
The pathogenesis and leukemia classification. **(A)** The development of leukemia involves various genetic abnormalities, including chromosome crossovers, deletions, and point mutations. **(B)** Abnormal proliferation of hematopoietic stem cells can lead to leukemia. Leukemia is divided into chronic leukemia and acute leukemia. Chronic leukemia is characterized by slow onset of peripheral blood or bone marrow cells, which can be divided into CML and CLL. A decrease in mature leukocytes and an impaired immune response may result in acute leukemia. Acute leukemia can be divided into AML and ALL.

Leukemia affects white blood cells. There are two main types of white blood cells: lymphoid cells and myeloid cells. Lymphocytic leukemia occurs when a lymphocyte becomes cancerous, and when bone marrow cells become cancerous, it is classified as myelocytic leukemia. Leukemia can be categorized as acute and chronic based on the growth rate of the cancer cells (Abhishek et al., 2023) (Figure 1). Acute leukemia refers to a fast-growing leukemia, while chronic leukemia refers to a slower-growing leukemia. Acute leukemias are more common and can occur at any age (Tebbi, 2021). In acute leukemia, the normal function of the bone marrow is disrupted, leading to the abnormal maturation and proliferation of immature cells. In acute leukemia, this can result in a decrease in the number of mature white blood cells, such as neutrophils, and impaired immune responses. Immature bone marrow cells may inhibit the function of antigen-specific T cells.

The humoral immune system is affected by the leukemia and the treatment, specifically for immunoglobulin G (IgG) and immunoglobulin M (IgM) which are the most affected immunoglobulins. It is also possible for patients in complete remission to have humoral deficits (Hansen et al., 2020). Chronic leukemia refers to a group of hematological malignant diseases characterized by a gradual onset, slow progression, and relatively well-differentiated naive cells in the peripheral blood and/or bone marrow. CLL is the most prevalent type of leukemia in adults. It is a lymphoproliferative disease marked by the proliferation of CD5(+) CD23(+) B cells, which are monoclonal and mature, in the peripheral blood, secondary lymphoid tissue, and bone marrow (Bosch and Dalla-Favera, 2019). The Philadelphia (Ph) chromosome is a result of a reciprocal translocation involving chromosome 9 and is now denoted as t (9; 22) (q34; q11). This abnormality carries a unique fusion gene known as BCR-ABL, which is currently considered a significant factor contributing to the chronic phase of CML (Goldman and Melo, 2003).

## 2 Leukemia treatment

Conventional treatment approaches for leukemia include chemotherapy, radiotherapy, and immunotherapy (Ci et al., 2022) (Table 1). Chemotherapy aims to kill leukemia cells and stop their proliferation, but it can harm the bone marrow and immune system due to its toxicity (McNeer et al., 2019; Gavillet et al., 2020; Alexander and Krull, 2021). Chemotherapy drugs encompass several classes of drugs, each with distinct mechanisms of action. Alkylating agents, such as bendamustine, play a pivotal role in leukemia therapy due to their ability to bind to DNA sites, inducing DNA double-strand breaks more effectively compared to other agents (Mahbub et al., 2019; Ci et al., 2022; Lalic et al., 2022). A study comparing bendamustine with phenylbutyrate mustard in Chinese CLL patients found that bendamustine had better therapeutic efficacy and significantly longer progression-free survival (Zhou D. et al., 2022). Additionally, antimetabolic drugs like thiopurines (e.g., 6-mercaptopurine) and cytarabine interfere with DNA replication and transcription, ultimately impeding tumor cell growth. Although 6-mercaptopurine is well tolerated, it may cause gastrointestinal (GI) toxicity in some patients, including hepatitis, hypoglycemia, nausea, and pancreatitis (Conneely et al., 2020). Cytarabine undergoes phosphorylation within cells, generating active triphosphate metabolites. These metabolites are then incorporated into DNA, disrupting cell cycle progression and triggering tumor cell death, particularly during the G1 to S phase transition (Di Francia et al., 2021).

**Table 1:** Leukemia treatment strategies and their characters.

Means of treatment	Medicine	Treatment process	Advantage	Limits	References
Chemotherapy	Bendamustine	Improved DNA penetration and localization, as well as more DNA double-strand breaks lasting longer	Better anti-tumor efficacy than classic products; Alkylating agent does not increase toxicity. Relatively well tolerated, and does not cause hair loss	Causes deep and prolonged lymphopenia, affecting both T and B-cell lineages	Ci et al. (2022), Lalic et al. (2022)
	6-mercaptopurine	Disruption of DNA replication and transcription processes induces cytotoxicity in cancer cells	Well tolerated	Gastrointestinal (GI) toxicity, including hepatitis, hypoglycemia, nausea, and pancreatitis	Conneely et al. (2020)
	Cytarabine	It activates intracellular metabolism through continuous phosphorylation to generate active triphosphate, which is incorporated into DNA as a pseudoprecursor instead of deoxycytidine triphosphate. It leads to cell cycle arrest from G1 phase to S phase and death of tumor cells	It can cross the blood–brain barrier, or it can be injected intrathecally	Myelosuppression; Leukopenia; Thrombocytopenia; allergy	Di Francia et al. (2021)
	Daunorubicin	Induced apoptotic and necrotic death of acute leukemia cells through changes in mitochondrial membrane potential (Δψm) and ROS generation, which promoted mitochondria membrane permeabilization and subsequent induction of apoptosis	High dose of daunorubicin significantly improved the survival rate of patients; Long-term effectiveness	Chemotherapy resistance	Al-Aamri et al. (2019), Dolatkhah et al. (2019), Al-Aamri et al. (2021), Jaime-Pérez et al. (2023)
	Doxorubicin	Causes DNA repair or induces cell death	Killing rapidly dividing cells and delaying the progression of solids and liquids	Cumulative dose can induce cardiotoxicity. Cardiac autonomic nervous system (ANS) dysfunction	Caru et al. (2019), Naci et al. (2019)
	Vincristine	Interferes with the assembly of microtubules in the mitotic spindle, leading to mitotic disruption and intermediate cell death	Alkaloids have been developed for various diseases such as anti-inflammatory, antibacterial and anti-tumor. It is injected in the form of intravenous fluids and can be used for various chemotherapy programs	Neurotoxicity; Hematological toxicity	Yang et al. (2021a), Dhyani et al. (2022)
	Dexamethasone	Influencing specific transcriptome programs and/or modulating early inflammatory responses associated with chemotherapy resistance may sensitize AML cells to chemotherapy-induced cell death; It has cytoplasmic and nuclear activity and can interfere with signal transduction or transcription factors	Improving disease-free and overall survival in hyperleukocytic AML patients	Affecting children’s sleep and behavior	Bertoli et al. (2018), Chougule et al. (2019), Rensen et al. (2020)
	Asparaginase	Consuming circulating asparagine and depriving cancer cells of amino acids	Asparaginase activity can measure be regularly monitored throughout the course of asparaginase therapy and can be a valuable tool to guide treatment management decisions	Hepatotoxicity; Hyperammonemia; Hyperglycemia; Hypersensitivity reaction; Hypertriglyceridemia; Coagulation diseases and thrombosis; pancreatitis	Salzer et al. (2018), Juluri et al. (2022)
Radiotherapy	/	It uses ionizing radiation (such as alpha, beta, or gamma rays) to kill cancer cells by directly irradiating malignant tissue	Reducing the size of the tumor Relieve compressive nerve pain caused by the tumor	Drowsiness, headache, fever, vomiting, exacerbation of pre-existing defects, dementia, leukoencephalopathy, secondary brain tumors	Michaelidesová et al. (2019), Li et al. (2024)
Immunotherapy	/	Active immune effector cells recognize appropriate tumor antigens and kill tumor cells	Patients who do not qualify for alloSCT and are in MRD status can be treated; Improved response rates and outcomes in patients with relapsed/refractory B-ALL	Harm the nervous system	Beyar-Katz and Gill (2018), Przespolewski et al. (2018), Inaba and Pui (2019)

Daunorubicin, classified as an anthracycline antibiotic, operates by inducing apoptosis and necrosis in acute leukemia cells through modulation of mitochondrial membrane potential and reactive oxygen species (ROS) production. While high doses of daunorubicin have shown significant improvements in patient survival, they carry the risk of inducing chemotherapy resistance (Al-Aamri et al., 2019; Dolatkhah et al., 2019; Al-Aamri et al., 2021; Jaime-Pérez et al., 2023). Similarly, doxorubicin, another anthracycline antibiotic, functions by either prompting DNA repair mechanisms or triggering cell death pathways. Its efficacy lies in targeting the rapidly dividing cells, thereby impeding the progression of both solid tumors and leukemias. However, prolonged use can lead to cardiotoxic effects, particularly in cumulative doses (Caru et al., 2019; Naci et al., 2019). Vincristine, classified as a vinca alkaloid, is widely utilized in leukemia treatment. It disrupts mitotic spindle assembly by interfering with microtubule formation, consequently inducing cell death (Yang Q. Y. et al., 2021; Dhyani et al., 2022).

Dexamethasone, recognized for its anti-inflammatory properties, exerts influence over specific transcriptome programming and early inflammatory responses implicated in chemotherapy resistance. It has demonstrated potential in sensitizing AML cells to chemotherapy-induced cell death by interfering with signal transduction pathways or transcription factors. While effective in managing inflammation-related conditions, its usage may impact children’s sleep patterns and behavior (Bertoli et al., 2018; Chougule et al., 2019; Rensen et al., 2020). A randomized controlled trial has shown that dexamethasone is more effective in treating children with ALL (Teuffel et al., 2011). Asparaginase constitutes a pivotal component of multidrug chemotherapy regimens for ALL in pediatric and young adult patients. By depleting circulating asparagine, it disrupts essential amino acid supplies to cancer cells. Monitoring asparaginase activity levels throughout treatment facilitates informed management decisions (Salzer et al., 2018; Juluri et al., 2022).

In the treatment of leukemia, radiation therapy is a common method. It uses ionizing radiation (such as alpha, beta, or gamma rays) to kill cancer cells by directly irradiating malignant tissue. By harnessing the targeting capabilities of specific materials, the radioactive isotopes could be direct to leukemia cells (Li et al., 2024). Antibodies with powerful targeting capabilities have been used to deliver radioisotopes to leukemia cells through the development antibody-isotope couplings (Jurcic, 2020). Radiotherapy is associated with significant side effects and can compromise host immunity (De Ruysscher et al., 2019). These effects may include drowsiness, headache, fever, vomiting, exacerbation of pre-existing conditions, dementia, leukoencephalopathy, and the development of secondary brain tumors (Michaelidesová et al., 2019). As the therapy outcomes improves, treatment toxicity becomes a major concern. Future directions may include reducing or eliminating testicular and intracranial radiation therapy (CRT) dose, and more effective and less toxic CNS-targeted drug therapy for patients should be developed (Chang et al., 2021).

Immunotherapy is a potential treatment for leukemia, and several forms of the treatment can be administered, including allogeneic bone marrow transplantation, therapeutic cancer vaccines, T-cell therapies, monoclonal antibody therapies, and donor lymphocyte infusion (Mu et al., 2023). Various immunotherapies employing T cells against AML encompass bispecific and dual antigen receptor-targeted antibodies, chimeric antigen receptor (CAR) T-cell therapy, and T-cell immune checkpoint inhibitors. These therapeutic strategies are continuously evolving with the goal of achieving potent anti-leukemic activity while minimizing T-cell cytotoxicity to healthy tissues (Daver et al., 2021). The mechanism involves the activation of immune effector cells that recognize tumor antigens and eliminate tumor cells (Beyar-Katz and Gill, 2018). Notably, immunotherapy offers advantages in treating patients with minimal residual disease (MRD) who are ineligible for allogeneic cell transplantation (Przespolewski et al., 2018). Additionally, it shows enhanced response rates and prognosis in patients with refractory B-cell acute lymphoblastic leukemia (B-ALL) (Inaba and Pui, 2019). To enhance the effectiveness of leukemia treatment, identifying specific targets and refining treatment regimens to minimize toxic side effects are essential (Tan et al., 2020).

While current ALL therapy is generally effective in children, it is highly toxic and might affect the function of various organs and systems (Ness et al., 2011). Ongoing trials involving new or current drugs to prevent and repair myocardial damage are developing to treat ALL disease and minimize drug side effects (Ness et al., 2011).

## 3 The relationship among gut microbes, probiotics, and leukemia

Recently, the alterations in certain species and metabolites of gut microbiota were identified to contribute to the development of leukemia (Ma et al., 2021). Gut microbiota consists of a large number of microorganisms that reside in the human gastrointestinal tract. They perform various important functions, including metabolism, immunity, and neural development. Increasingly, gut microbiota are believed to play a significant role in health and disease (Zhou Y. et al., 2022). The gut microbiota could possibly influence the occurrence and development of leukemia by regulating immune cells, promoting inflammation, causing infection from pathogenic bacteria, affecting metabolites, and influencing overall metabolism and gene mutation.

The occurrence, treatment, and prognosis of various tumors are closely linked to gut microbiota, and gut microbial metabolites often play a mediating role in this relationship (Jaye et al., 2022). Among the metabolites, bile acids are particularly important as they are key players in maintaining the normal physiological functions of the human body. Chenodeoxycholic acid collaboratively promotes the accumulation of lipid droplets and lipoperoxidation through the ROS/p38 MAPK/DGAT1 pathway. It also inhibits the polarization of M2 macrophages and suppresses the progression of AML (Liu et al., 2022). Disrupting amino acid metabolism has been an effective treatment for leukemia. Leukemia cells have a high demand for amino acids to support their increased biosynthetic needs, making blocking amino acid uptake and utilization a promising approach in both preclinical and clinical studies (Chen and Zhang, 2024). In AML cells, vitamin C has been found to enhance TET2 activity and mimic its restoration. Restoring TET2 activity creates a vulnerability in leukemia cells, making them more responsive to poly ADP ribose polymerase inhibitors (PARPi) (Bedhiafi et al., 2022).

When the imbalance of the gut microbiota disrupts the intestinal epithelial barrier, some gut microbes may enter the bloodstream or nearby lymph nodes, triggering inflammatory immune responses via metabolic disturbances, immune cell activation, and alterations in crucial intracellular signaling pathways, potentially leading to cancer development. Probiotics offer health advantages to the host by ameliorating intestinal dysbiosis, enhancing nutrient absorption, safeguarding the intestinal mucosal barrier, modulating immunity, and inhibiting intestinal inflammation (Zhou Y. et al., 2022). Prebiotics and probiotics can modulate gut microbiota which might affect leukemia. The role of prebiotics in the treatment and development of leukemia is still uncertain, although inulin has shown promise in treating leukemia (Martyniak et al., 2023). Certain probiotics, such as lactic acid bacteria, have demonstrated anti-cancer activity in the laboratory (Garbacz, 2022). The stability of the gut microbiota is beneficial for leukemia patients, and it is crucial to explore methods for maintaining a balanced gut microecology for leukemia patients (Huang, 2022). Gut microbiota play a significant role in both the benign and adverse effects linked with the development of hematological tumors (Table 2).

**Table 2:** Effects of gut microbes on leukemia patients.

Species	Type of research	Classification	Implications in leukemia treatment	References
*Lactic acid bacteria*	*in vitro*	Gram-positive bacteria	Regulation of epithelial tight junction protein to improve intestinal barrier function	Uribe-Herranz et al. (2021), Garbacz (2022)
*Faecalibacterium prausnitzii*	*in vitro*	Gram-positive bacteria	Produces butyrate, improves intestinal barrier	He et al. (2021)
*Bifidobacterium*	*in vitro*	Gram-positive bacteria	Modulate the hosts’ immune system via Immunoglobulin E (IgE) production, maintenance and improvement in the Th1/Th2 balance	Kaźmierczak-Siedlecka et al. (2021), Mandelbaum et al. (2023)
*Lactobacillus rhamnosus*	*in vitro*	Gram-positive bacteria	Increases the production of Immunoglobulin A (IgAs), protects against local inflammation	Banna et al. (2017), Gu et al. (2021)
*Lactobacillus plantarum*	*in vitro*	Gram-positive bacteria	Produces metabolites that are selectively cytotoxic to cancer cells but not to healthy cells	Chuah et al. (2019)

There exist beneficial bacteria capable of mitigating disease progression and stabilizing the patient’s immune system. For instance, lactic acid bacteria enhance intestinal barrier function *in vitro* by modulating epithelial tight junction proteins (Uribe-Herranz et al., 2021; Garbacz, 2022). *Faecalibacterium prausnitzii* produces butyrate, which aids in improving the intestinal barrier (He et al., 2021). Butyrate has anti-inflammatory effects and enhances intestinal barrier and mucosal immune function by modulating signaling pathways involved in nuclear NF-κB and inhibiting histone deacetylase (Wang et al., 2022). *Bifidobacterium bifidum* has been utilized in leukemia treatment to sustain and homeostatically modulate the host’s immune system by promoting the production of Immunoglobulin E (IgE) (Kaźmierczak-Siedlecka et al., 2021; Mandelbaum et al., 2023). In a study, participants who consumed *Bifidobacterium bifidum* showed decreased nitroreductase activity in their feces and increased β-glucosidase activity. The elevated levels of β-glucosidase could be seen as a beneficial aspect for health (Wollowski et al., 2001). Furthermore, *Lactobacillus rhamnosus* GG (LGG) can prevent local inflammation by enhancing immunoglobulin A (IgA) production (Banna et al., 2017; Gu et al., 2021). LGG can promote the differentiation and development of B cells in the lamina propria of the piglets’ intestine and the production of IgA. Furthermore, LGG activates the EGFR/AKT/NF-κB pathway via the p40 protein, which stimulates porcine intestinal epithelial cells (IPEC-J2) to secrete a proliferation-inducing ligand (APRIL). This process further boosts the production of IgA by B cells, thereby enhancing intestinal immunity (Jin et al., 2021). Metabolites produced by *Lactobacillus plantarum* were found to have selective, time- and dose-dependent cytotoxic effects on cancer cells without toxic effects on healthy cells in studies on leukemia and breast cancer cell lines (Chuah et al., 2019).

**Figure 2:**
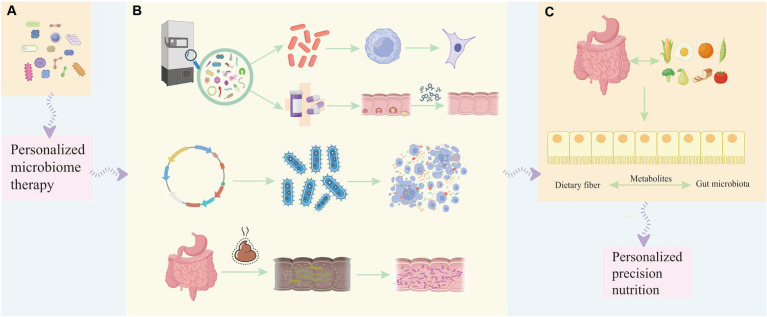
The involvement of probiotics in different stages of tumor development helps in personalized treatment and prognosis of the disease. **(A)** Single strains or combinations of strains can be used as diagnostic and early-stage biomarkers to help determine the occurrence or development of tumors, and personalized microbiome therapy can be applied. **(B)** During the treatment period of leukemia, the beneficial bacteria selected from the bacterial strain library can not only regulate the proliferation and apoptosis of tumor cells at the cellular level, but can also be used in conjunction with chemotherapy drugs to improve the effect of chemotherapy and reduce the adverse reactions caused by chemotherapy. Engineered probiotics can improve the efficacy of immunotherapy in mouse tumor models through metabolic regulation of the tumor microenvironment. Fecal microbiota transfer (FMT) has been developed to restore diverse microbial communities lost during subsequent treatment in patients with acute myeloid leukemia, thereby suppressing, reducing or even preventing treatment-related complications. **(C)** Therapeutic benefits to the prognosis of cancer patients by modulating dietary and balanced nutritional intake and enhancing the gut microbiota through dietary fiber-gut microbiota related mechanisms affecting the patient’s cancer treatment response to immunotherapy.

Probiotics are therapeutic formulations that contain identified, propagated, or engineered probiotics, aiming to provide health benefits or enhance standard care (Singh et al., 2023). Various beneficial mechanisms of probiotics for the prevention and treatment of human diseases have been identified, including the regulation of gut microbiota, strengthening of the intestinal barrier, protection of the intestinal epithelium from pathogenic invasion, and enhancement of the immune system (Li et al., 2021; Lu et al., 2021). Hence, specific probiotic strains may have a significant impact on cancer prevention, potentially through the regulation of gut microbiota and immune response, suggesting their potential use in cancer prevention as well as adjunct therapy alongside anti-cancer chemotherapy (Śliżewska et al., 2020; Liu et al., 2021). The specific composition of the gut and tumor microbiota may affect the bioavailability of administered drugs and the host’s immune environment, thereby influencing patient prognosis. Personalized therapies that improve treatment efficacy by supporting and manipulating the microbiota are an emerging area in cancer treatment (Figure 2).

### 3.1 Biomarkers associated with leukemia

Leukemia is a group of hematologic malignancies derived from bone marrow stem cells. Leukemic stem cells undergo abnormal and poorly regulated proliferative processes, induced by genetic mutations. It has been shown that CD123 is an important marker for identifying and targeting refractory or recurrent leukemic stem cells (Shi et al., 2019). MicroRNAs are considered biomarkers of tumor and disease in hematologic malignancies. Cyclin-dependent kinase 6 (CDK6) is targeted by some miRNAs, such as miR-29b, miR-218, miR-582, and miR-187. High expression of CDK6 can significantly reduce the overall survival rate of AML patients, which suggests that CDK6 may have the potential prognosis and treatment value of AML (Liu et al., 2020).

### 3.2 Probiotics participate in the body’s immune response

Probiotics could influence the production of anti-inflammatory cytokines, activate phagocytosis, and regulate tumor cell proliferation and apoptosis in the treatment of hematological tumors (Huang et al., 2023). *Bifidobacterium bifidum* exhibits a cytotoxic effect on tumor cells, reducing their proliferation by inhibiting growth factor signaling, and promoting mitochondria-mediated apoptosis (Badgeley et al., 2021). Various *Lactobacillus* species possess different degrees of antiproliferative activity against the K562 cell line (Tuo et al., 2015). However, the exact molecular mechanism has not been revealed. Moreover, metabolites obtained from *Streptomyces* fermentation broth can exert cytotoxic effects on the K562 cell line (Zhang et al., 2022).

Probiotics influence cell activity in other human leukemia cell lines through their impact on cellular metabolism or associated immune response factors. For example, *Levilactobacillus brevis* JCM 1059 binds to CAP-1 in the human leukemia monocytic cell line THP-1 cells, which plays a crucial role in bacterial uptake and subsequent production of the pro-inflammatory factor interleukin-12 (IL-12) in THP-1 cells (Yin et al., 2023). *Bacillus subtilis* could induce the production of the anti-inflammatory factor IL-10 and the pro-inflammatory factor IL-12 in THP-1 dendritic cells (THP-1 DC) (Uesugi et al., 2023). Certain *Lactobacillus plantarum* strains could trigger selective cytotoxic effects and have the ability to induce apoptosis in human leukemia cells HL60 while preserving normal cells (Chuah et al., 2019). In light of the anti-apoptotic nature of tumor cells and the inflammatory molecules associated with tumor cell development, the immune response elicited by probiotics against pathogenic bacteria or tumor cells has shown a positive impact on the immune system.

The gut microbiota is associated with the responses to immune checkpoint inhibitors (ICIs), anti-PD-1/PD-L1, or anti-CTLA-4 treatments. Probiotics, particularly *Lactobacillus* and *Bifidobacterium*, hold the potential for the prevention and treatment of various cancer types (Singh et al., 2023). *Bifidobacterium longum* can activate CD4^+^ T cells and enhance Th1 responses, thereby priming CD8^+^ T cells upon their interaction with dendritic cells (DCs). The DNA of *B. longum* contains immunostimulatory motifs that are capable of activating innate immunity. Evaluation of three *B. longum* strains found their functions in the activation of DCs in Peyer’s patches, suggesting a potential antigen-independent antitumor activity mediated through innate immunity. Mice with significantly higher levels of *Bifidobacterium bifidum* were found to have slower tumor progression and better response to anti-PD-1 treatment in a study on melanoma (Routy et al., 2018). This suggests that modification in the gut microbiome may improve the efficacy of certain chemotherapy drugs and reduce their toxicity (Sági et al., 2022). Lactic acid bacteria have demonstrated anti-cancer properties through various mechanisms (Yuan et al., 2023). For example, intra-nasal administration of live cells of *Lactobacillus casei* BL23, induced by human papilloma virus (HPV), led to reduced tumor growth in a model. *In vitro* experiments with *Lactobacillus reuteri* BCRC14652 showed destruction of the cell membrane of colon cancer HT29 cells, inhibition of tumor necrosis factor (TNF) induced NF-κB activation, and suppression of cancer cells growth through apoptosis. The supernatant of *Lactobacillus reuteri* ATCC 6475 (Lr-S 6475) can affect the apoptosis signaling pathway activated by TNF in myeloid leukemia cells (Iyer et al., 2008).

Furthermore, extracellular polysaccharides (EPS), peptidoglycan, nucleic acid, bacteriocin, and S-lamin produced by lactic acid bacteria have been shown to inhibit the growth of cancer cells. EPS produced by *Lactobacillus acidophilus* and *Lactobacillus rhamnosus* directly induce the activity of Beclin-29 (an autophagy protein) and GRP1 (an endoplasmic reticulum chaperone), resulting in the inhibition of HT-78 cell growth. They also indirectly regulate apoptosis by stimulating Bcl-2 (B-cell lymphoma 2) and the pro-apoptotic gene Bak of the Bcl-2 family. Additionally, the combination of *Lactobacillus acidophilus* and *Lactobacillus casei* with 5-FU induced apoptosis of LS513 cancer cells, suggesting that these species could be used as adjuvants for anticancer chemotherapy. Overall, lactic acid bacteria have displayed enhanced cytotoxicity against human chronic myelogenous leukemia K562 cells and colorectal tumor HCT116 cells (Dicks and Vermeulen, 2022), highlighting their potential as an adjunct to drug treatment of leukemia.

An engineered probiotic *Escherichia coli* Nissle 1917 has shown promising results in mouse tumor models by increasing L-arginine concentrations, enhancing the number of tumor-infiltrating T cells, and synergistically working with PD-L1 blocking antibodies to clear tumors. It suggests that the metabolic modulation of the tumor microenvironment by engineering probiotics could enhance the efficacy of immunotherapy (Canale et al., 2021). While transgenic T-cell therapy (CAR-T) has demonstrated significant effectiveness in treating certain B-cell-driven hematologic malignancies, the complex immune evasion mechanisms and tumor microenvironment pose limitations to its efficiency (Huang et al., 2022). However, combining probiotic therapy with CAR-T cell therapy using synthetic gene circuits could detect and respond to synthetic CAR targets delivered by colonizing probiotics in solid tumors. This approach expands the potential of CAR-T cell therapy (Vincent et al., 2023), which highlights the potential benefits of combining engineered probiotics with existing therapeutic approaches to improve treatment outcomes in cancers.

### 3.3 Probiotics are involved in regulating homeostasis the of gut microbiota

**Figure 3:**
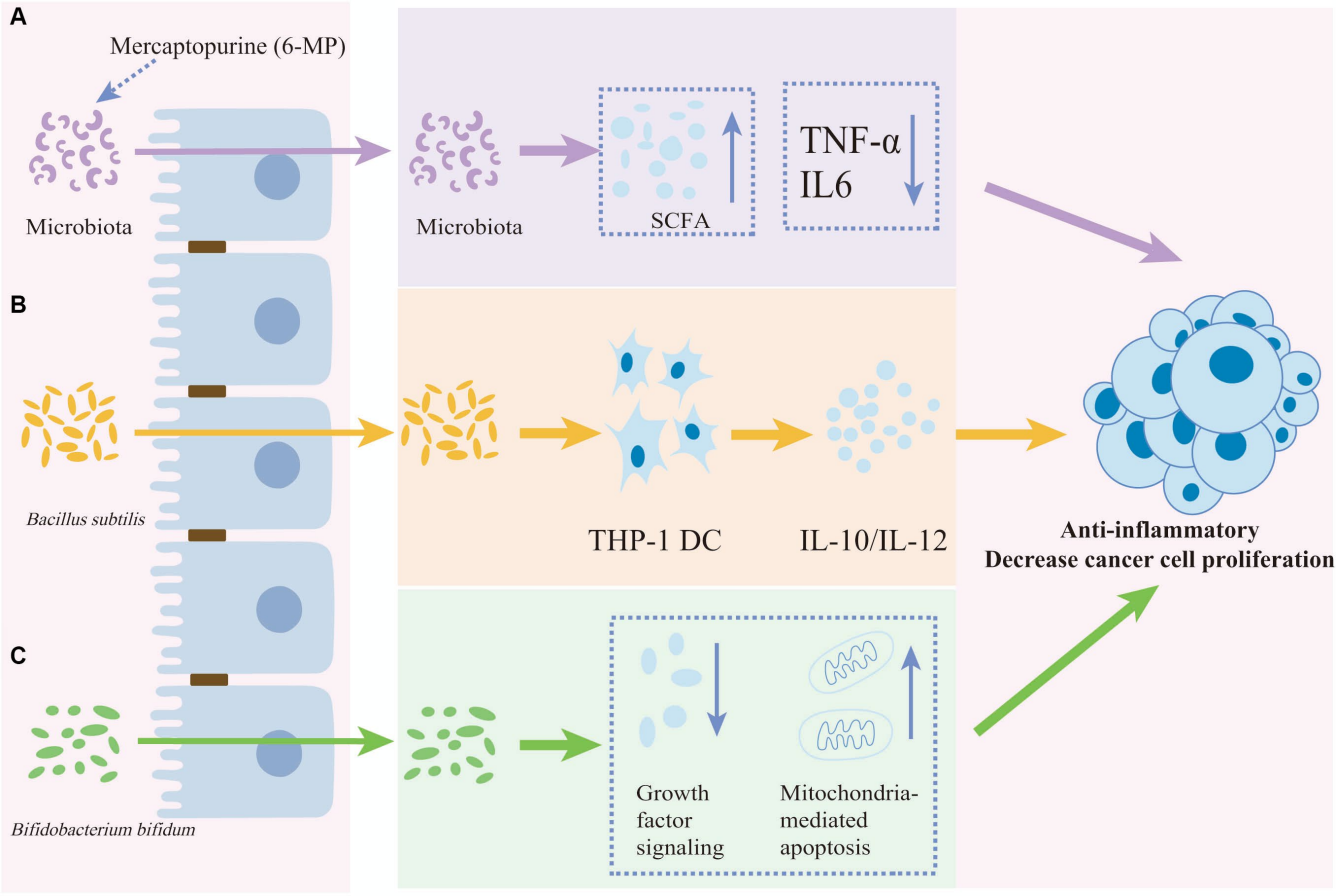
The anti-inflammatory and anti-proliferative effects of anti-cancer drugs or probiotics. The anti-inflammatory and decreasing cancer cell proliferation effects of anti-cancer drugs or probiotics on cancer cell. **(A)** The anti-cancer effect of mercaptopurine potentially functions by reducing IL-6 and TNF-α levels and increasing short-chain fatty acids (SCFAs) mediated by the gut microbiota. **(B)** *Bacillus subtilis* induces both the anti-inflammatory factor IL-10 and the pro-inflammatory factor IL-12 in THP-1 dendritic cells. **(C)** *Bifidobacterium bifidum* reduces cancer cell proliferation by inhibiting growth factor signaling and promoting mitochondria-mediated apoptosis proliferation.

Gut microbes can participate in stabilizing host immune cell populations, and interactions between the microbiome and the immune system contribute to protecting the host against a range of diseases, including cancer (Sarkar et al., 2024). Probiotics have been found to have potential benefits for leukemia patients by regulating the immune stability of the gut microbiota (Figure 3). The relative abundance of *Actinobacteria*, *Acidobacteria*, and *Chloroflexi* in myeloid leukemia patients increased at phylum-level, and *Streptococcus* increased at genus level. Species enriched in myeloid leukemia patients comprised *Sphingomonas*, *Lysobacyer*, *Helicobacter*, *Lactobacillus*, *Enterococcus*, and *Clostridium sensu stricto* (Yu et al., 2021). Children with leukemia had significantly reduced levels of *Lactobacillus* and *Bifidobacterium* as well as *E. coli* strains in the intestinal tract compared to healthy children (Huang et al., 2012). The impact of probiotics extends beyond their ability to colonize in the intestine, they also affect the functional integrity of the mucosal epithelial barrier and the immune cells responsible for maintaining its integrity (Wieërs et al., 2019). LGG produces antimicrobial substances that compete with pathogenic bacteria for adhesion to the epithelium. Moreover, it increases mucosal IgA production and inhibits toxin production (Vivarelli et al., 2019). Butyrate produced by *Enterococcus faecalis* has been shown to inhibit the absorption of lipopolysaccharide by epithelial cells and repair the damaged intestinal barrier in mice with AML, thereby delaying the development of AML (Wang et al., 2022). Levels of *Lacertococcaceae* and *Peptococcus* were correlated with changes in the levels of intestinal short-chain fatty acids (SCFAs) such as butyrate. In addition, the levels of both were negatively correlated with pro-inflammatory factors such as IL-6 and tumor necrosis factor-α (TNF-α) (Jin et al., 2023). This suggests that probiotic-induced anti-inflammatory responses play an important role in maintaining intestinal immune stability and improving ALL progression. Furthermore, acetate, which is secreted by *Escherichia coli* KUB-36 as a major metabolite, exerts anticancer effects by suppressing IL-6, IL-1β, and TNF-α. It enhances the anti-inflammatory activity of macrophages, thus promoting homeostasis in the intestinal internal environment (Nakkarach et al., 2021).

Probiotics help maintain the immune stabilization of the gut microbiota and can attenuate the effects of leukemia complications. As previously described, one common complication of leukemia is bloodstream infection (BSI) caused by a compromised intestinal microenvironment (Silva et al., 2022). Changes in the gut and fecal microbiome in a pediatric T-ALL mouse model, SCFA levels, and the gut barrier may lead to bacterial translocation and subsequent BSI. SCFAs supplementation was found to improve the effects of BSIs indicating that rational probiotic supplementation can help maintain the stability of the intestinal microenvironment and thus alleviate leukemia-induced BSI (Song et al., 2022). Graft-versus-host disease (GVHD) is another common complication of leukemia treatment (Choe and Ferrara, 2021). Patients with GVHD inhibit the expression of the antimicrobial peptide alpha-defensin, which leads to a reduced diversity of the gut microbiota. Evaluation of the bacterial composition of feces from patients after hematopoietic stem cell transplantation showed that an increase in bacterial diversity, particularly in the number of *Brunswickia* species, was significantly associated with a reduction in GVDH mortality. This suggests that supplementation with specific beneficial gut bacteria could be effective in reducing the impact of GVHD in patients (Jenq et al., 2015). Probiotics may affect the mechanism of GVHD by up-regulating genes associated with immune response through the gut metabolite indole. This can help limit gut epithelial damage, reduce the production of inflammatory cytokines, and decrease the severity and mortality of GVHD (Swimm et al., 2018; Ma et al., 2021).

Fecal microbiota transplantation (FMT) and other strategies aim to restore various microbes that are lost during disease treatment, which can help prevent complications in AML patients (Malard et al., 2021). Additionally, ICIs are used to activate the suppressed immune system and enhance the elimination of cancer cells. Colonization resistance is a physiological function that defends against colonization and infection by pathogenic bacteria (Liao et al., 2024). FMT can also restore colonization resistance and potentially eliminate multidrug-resistant pathogens, offering promising results in eradicating recurrent *C. difficile* infections (van Nood et al., 2013). However, while probiotics are beneficial for internal gut homeostasis, FMT is more effective as it re-introduces the entire flora into the gut. It is important to carefully assess the microbiota beforehand to prevent the spread of pathogens, especially in immunocompromised individuals. Combining probiotic therapy with FMT may provide better outcomes for patient recovery.

### 3.4 Combinational chemotherapy with probiotics

Chemotherapy is often necessary for leukemia patients, but it can disrupt the balance of gut microbiota and compromise the protective function of the intestinal barrier. This disruption of the gut microbiota can impact the effectiveness of chemotherapy (Cong et al., 2019). Gut microbes can interact with chemotherapeutic agents through transport, immunomodulation, metabolism, enzymatic degradation, reduction of microbial diversity, and ecological changes (Alexander et al., 2017). The disturbance of the gut microbiota can lead to various inflammatory reactions, which in turn can affect cell and immune function, ultimately resulting in weakened immunity (Huang, 2022). Therefore, it is crucial to maintain the balance of gut microbiota in chemotherapy patients to improve efficacy and reduce its toxicity. One approach is to target the gut microbiota by supplementing with intestinal probiotics or using probiotic drugs. This can play an important role in improving the effectiveness of chemotherapy and alleviating its adverse effects (Reyna-Figueroa et al., 2019).

Cyclophosphamide is a commonly used drug in leukemia chemotherapy regimens. Antibiotics targeting gram-positive bacteria significantly reduce the efficacy of CP (Wang et al., 2019), suggesting that gram-positive bacteria, such as *Enterococcus* and *Lactobacillus*, are key in modulating the anti-tumor efficacy of CP against hematological tumors. These bacteria have the capacity to enhance the anti-tumor effect of CP by modulating the CP-mediated aggregation of Th1 and Th17 cells (Viaud et al., 2013). Oral mucositis and oral health are prevalent side effects of treatment. Specific probiotics can lower the occurrence of severe oral mucositis by enhancing the growth and safeguarding bacterial populations, leading to a decrease in adverse reactions, severity, and frequency (Rodriguez-Arrastia et al., 2021). Probiotics supplementation can alleviate symptoms of chemotherapy-induced chronic gastrointestinal inflammation and diarrhea (Reyna-Figueroa et al., 2019). 5-Fluorouracil (5-FU) is one of the most widely used chemotherapies for the treatment of malignant tumors (Gibson et al., 2013). However, some patients treated with this drug experience gastrointestinal side effects. 5-FU induces apoptosis and inhibits enterocyte proliferation via TNF-α, resulting in the weakening of the epithelial barrier function and subsequent inflammation. A combination of a broad-spectrum antimicrobial, ampicillin, and a gram-negative bacteria antibiotic drug, aztreonam, can significantly reduce the severity of intestinal mucositis and inhibit the influx of inflammatory factors (Hamouda et al., 2017).

Probiotics can enhance the effectiveness of chemotherapy when used in combination with chemotherapeutic agents for the treatment of malignant tumors. For example, oral administration of *Lactobacillus acidophilus* along with cisplatin to lung cancer mice reduced tumor volume and increased survival time in the mice. This suggests that supplementation with probiotics could enhance the anti-tumor response by up-regulating the expression of IFN-γ and Prf1 (Bingula et al., 2018). Similarly, in a rat model of colorectal cancer, a probiotic mixture containing *Lactobacillus lactis* and two *B. bifidum* strains improve the therapeutic effectiveness of 5-FU chemotherapy, potentially reducing tumor malignancy (Sivan et al., 2015). Furthermore, the insight into the relationship between the chemotherapeutic drug irinotecan and the gut microbiota reveals how the microbiota affects chemotherapy efficacy and toxicity through microbial ecological leapfrogging, microbial enzyme catalysis, and immunomodulation (Yue et al., 2021). Probiotics and their engineering interventions, other microbial strategies, and dietary interventions have been used to enhance chemotherapy efficacy and decrease toxicity (Yue et al., 2021). The efficiency of cyclophosphamide is significantly influenced by gut microbiota. *Lactobacillus johnsonii* and *Enterococcus sheila* have the ability to migrate to the spleen, where they activate the helper T cell 1 (Th1) and helper T cell (Th17) immune responses, thereby enhancing the treatment responses to cyclophosphamide (Chrysostomou et al., 2023). These findings highlight the potential for microbiota manipulation as a personalized approach to cancer therapy. However, more in-depth studies are needed to explore the use of probiotics in combination with chemotherapeutic agents for mitigating disease progression in leukemia models.

### 3.5 Probiotics can improve the adverse effects of treatments

During cancer treatment, it is common for healthy cells, especially those with a high rate of cell division, to be affected, leading to undesirable side effects. Many cancer drugs have the unintended effect of inducing apoptosis in healthy cells in the gastrointestinal tract, causing mucosal damage. IL-10 is a crucial cytokine that blocks inflammatory responses and prevents excessive immune responses. *Bifidobacteria* not only reduce pro-inflammatory cytokines, but also increase levels of anti-inflammatory cytokines such as IL-10 (Imaoka et al., 2008). Probiotics attenuate intestinal histopathologic changes induced by certain drugs in cancer treatment (Badgeley et al., 2021). Probiotics can mitigate histopathological changes caused by certain drugs in cancer treatment. They help restore the normal structure and function of the gastrointestinal wall in experimental animals, and alleviate the changes in gastrointestinal permeability that typically occur during mucositis (Olazagoitia-Garmendia et al., 2024). Furthermore, probiotics can also counteract the loss of mucin-secreting goblet cells, or regulate the expression of genes that encode mucin-secreting, ensuring the proper functioning of these cells. In addition, probiotic treatment can improve the intestinal immune barrier by enhancing the production of intestinal IgA (López-Gómez et al., 2023).

A study investigated the impact of probiotic supplementation on chemotherapy-induced gastrointestinal side effects in children with acute leukemia. The finding revealed that three out of eight gastrointestinal side effects (nausea, vomiting, and bloating) were significantly reduced in the acute leukemia patients who received probiotics. Additionally, daily supplementation with *L. rhamnosus* effectively lowered chemotherapy-induced gastrointestinal side effects in the children (Reyna-Figueroa et al., 2019). In another study focusing on the effects of lactate synbiotics administration on chemotherapy-induced diarrhea (CID), nausea, vomiting, and constipation in children with ALL undergoing maintenance chemotherapy, the incidence of constipation was significantly lower in the group that received lactate synbiotics compared to the control placebo group. The use of synbiotics supplements was found to effectively reduce CID in patients and can be considered as a simple and effective approach to alleviate CID in patients with ALL (Eghbali et al., 2023; Zhang et al., 2023). A meta-analysis on the use of probiotics for the prevention and treatment of CID found that probiotics administration can effectively reduce the levels of serum endothelin (ET), Diamine oxidase (DAO), D-lactic acid, and TNF-α. This helps decrease the permeability of the intestinal mucosa and protect and repair the intestinal mucosal barrier function (Lu et al., 2019).

The microbiota undergoes changes during the onset and treatment of leukemia, and these changes may affect anti-leukemia treatment and prognosis. Restoring the altered microbiota may improve patient outcomes (Zhou Y. et al., 2022). Probiotics may help manage the side effects of chemotherapy and radiotherapy for acute leukemia, especially in reducing the infection risk and promoting intestinal recovery after treatment-induced injury while restoring normal function (Martyniak et al., 2023). Probiotics exert their beneficial effects through four different mechanisms: competing with pathogens for nutrients and adhesion sites, improving the barrier function of the intestinal lining, regulating the immune system and producing neurotransmitters such as gamma-aminobutyric acid (GABA), and serotonin (Sánchez et al., 2017). The gut microbiota changes throughout the progression of the disease, and certain probiotics have been found to have a positive impact (Wieërs et al., 2019; Huang et al., 2023). For example, *Lactobacillus* and *Bifidobacterium* can interact with proteins that regulate the cell cycle, thereby inhibiting the proliferation of cancer cells which are often resistant to apoptosis. *Lactobacillus* and *Bifidobacterium* can overcome this resistance by activating pro-caspase and down-regulating anti-apoptotic Bcl-2 and up-regulating pro-apoptotic Bax proteins (Nowak et al., 2019). Modifying diet, probiotic substances, FMT, and antibiotic restoration can enhance the gut microbiome, potentially providing therapeutic benefits for the prognosis of cancer patients (Gately, 2019).

Cancer-induced nutritional deficiencies and nutritional adverse effects may create a vicious cycle that is detrimental to the treatment and prognosis of patients (Trujillo et al., 2018). The influence of dietary intake on the response to cancer treatment, particularly immunotherapy in melanoma patients, has been attributed to the mechanisms involving dietary fiber and gut microbiota (Spencer et al., 2021; Yang Y. et al., 2021). Understanding how diet affects the structure and function of microbiota during cancer treatment, as well as the role of microbiome modification in nutrient and drug metabolism, can guide the development of a precise nutritional approach for cancer treatment (Greathouse et al., 2022; Wei et al., 2022). The gut microbiota can influence gene–environment interactions, especially dietary interactions (Org et al., 2015). Furthermore, prudent dietary patterns can significantly affect the presence of certain bacteria, such as *F. nucleatum*, which has been shown to interact with the genetic characteristics of tumors (Hamada et al., 2018). Therefore, the use of personalized precision nutrition tailored to each patient’s needs can help regulate the balance of gut microbiota and minimize the adverse effects of drugs, ultimately improving patient prognosis. Probiotics have demonstrated effectiveness in fighting various cancers and lowering the risk of cardiovascular disease. It is crucial to reveal the complex interactions among probiotics, the gut microbiota, and diverse physiological systems, highlighting the importance of conducting additional clinical trials (Naeem et al., 2024).

## 4 Application of the zebrafish model in leukemia treatment

Zebrafish are a valuable animal model for leukemia research due to their low feeding costs, short spawning cycle, high numbers, capability of *in vitro* fertilization, transparent embryos, close similarity to the human genome, and remarkably conserved blood system (Robertson et al., 2016; Tanguay, 2018). In contrast to other animal models, the *in vitro* fertilization and embryonic transparency of zebrafish allow for direct use in live imaging and are easily manipulated. This facilitates the visualization and study of specific cell lineages and the monitoring of leukemia initiation and progression in live zebrafish (Harrison et al., 2016). Especially, in the early stages of development, zebrafish lack a functional adaptive immune system, thus it is easy to study their innate immune system in the absence of an adaptive immune system. This feature allows for the establishment of hematological tumor xenograft models to explore the efficacy and toxicity of different anti-leukemia drugs during the zebrafish embryonic period (Page et al., 2013). The optical transparency of zebrafish enables *in vivo* imaging, making the reverse genetic characterization of CRISPR-Cas9 gene editing technology a widely utilized approach in zebrafish disease models (Cong et al., 2013).

Human leukemia genes can be integrated into the zebrafish genome by CRISPR-mediated double-strand breaks and sequence donors specifically designed based on DNA repair mechanisms (Haapaniemi et al., 2018). For example, microinjection of the human mixed lineage leukemia 1 (MLL1)-AF 9 fusion gene normally results in AML, and microinjection of its mRNA into zebrafish embryos recapitulates the myeloproliferative disorders observed in murine models and in human disease (Tan et al., 2018). A T-ALL transgenic zebrafish model was used to determine the effectiveness of leukemia in eliminating immature T cells in developing zebrafish, suggesting the utility of zebrafish models for antitumor drug candidate identification and providing a new approach for targeted leukemia treatment (Ridges et al., 2012). Gut microbes have the ability to regulate immune cell activity and inflammation-related factors thereby influencing the immune system. Similar findings have been observed in zebrafish experiments, where intestinal microbiota regulates intestinal tumor necrosis factor receptors and alkaline phosphatase to maintain normal levels of neutrophils. This will facilitate the alleviation of intestinal inflammation induced by leukemia treatment (Bates et al., 2007).

L-asparaginases can be used for consolidation therapy in the induction phase of ALL. One study revealed very similar activity to Leucoginase by injecting zebrafish with recombinant *Lactobacillus* L-asparaginase I. Toxicity studies have indirectly demonstrated that *Lactobacillus* L-asparaginase is a better choice for the treatment of ALL (Suresh et al., 2020). Additionally, supplementation of zebrafish with SCFAs after tail trauma has been shown to reduce the recruitment of pro-inflammatory cells and neutrophils to the wound (Cholan et al., 2020). These findings lay the foundation for further investigation into the association between gut microbiome dysbiosis and the development of leukemia. A zebrafish T-ALL model was employed to evaluate drug-induced cell death in zebrafish ALL using a high-throughput screening platform. The effectiveness of high-throughput drug screening in detecting potent synergistic effects between novel T-ALL drug combinations involving AKT/mTORC1 inhibitors and dasatinib (a broad-spectrum tyrosine kinase inhibitor) (Laukkanen et al., 2022). The establishment of zebrafish models suggest that the impact of probiotic drugs on zebrafish leukemia models might be possible. The exploration of zebrafish models for mimicking human diseases, leveraging the CRISPR-Cas system and the Tol2 transposon system, has demonstrated the potential of both genetically engineered and induced zebrafish leukemia models in studying the genetic heterogeneity of leukemias and advancing related drug discovery. However, these models remain limited in their contribution to the future of precision therapy for leukemia patients, primarily due to the high complexity of recurrent mutations and the aggressive nature of leukemias (Yi et al., 2022).

The spatial distribution and coherence of bacterial strains in zebrafish exhibit significant variability, suggesting the potential for targeted manipulation and control of gut microbiota in specific regions. This manipulation could potentially help alleviate symptoms related to leukemia treatment (Zhong et al., 2022). Zebrafish have become a popular model for studying both normal and malignant blood cell development in humans, as well as for screening drugs related to blood disorders (Ye et al., 2019). Modifying the gut microbial composition in zebrafish can influence the functions of intestinal cells not only in terms of cellular metabolism but also the nervous system and bone health (Carnovali et al., 2021). The leukemia model of zebrafish offers new insights for probiotics-assisted treatment of leukemia. Additionally, using zebrafish disease models to understand the relationship between probiotics and the prognosis of leukemia treatment may help unravel the mysteries of curing leukemia.

Probiotics can help maintain a healthy balance of microorganisms in the intestines, which can potentially serve as an auxiliary means to prevent and alleviate adverse drug reactions (Ren et al., 2022). The gut microbiome could impact immune regulation, reduce inflammation, and restore intestinal homeostasis. Monitoring changes in gut microbial diversity and composition before and after treatment can be useful in predicting the occurrence of adverse reactions to chemotherapy (Xia et al., 2023). Thus, probiotics can be used in conjunction with anti-cancer drugs to help reduce inflammation levels (Sharma et al., 2021). Personalized therapeutic strategies should consider individual variations in gut microbiota, as well as the phenotypes and genotypes of hematologic malignancies. This approach involves meticulous drug sensitivity testing and customized nutritional plans tailored to create optimal conditions for effective dietary and probiotic supplementation, aiming to reduce gastrointestinal side effects and reinforce the immune barrier in hematologic malignancies (Song et al., 2020). A quantitative experimental framework that maps the human microbiome’s ability to metabolize small molecule drugs, known as microbiome-derived metabolism-screen, has been developed (Javdan et al., 2020). This study identifies interactions between drugs and the microbiome among different individuals, highlighting the potential for using the gut microbiome in drug development and personalized medicine. These findings provide new insights into the role of the microbiome and probiotics in personalized treatment of leukemia (Wen et al., 2019).

## 5 Perspectives

Currently, it remains crucial to investigate the underlying mechanisms through which probiotics can contribute to the treatment of leukemia. It is important to thoroughly examine the relationship between probiotics and leukemia. Thus, we can potentially develop more precise and targeted therapeutic approaches, such as specific probiotic supplements, microbiome transplants, and dietary interventions that promote beneficial microbial populations. This focused approach can be more effective than non-targeted probiotic supplements and may help reduce leukemia incidence and eliminate the need for anti-leukemia treatments with long-term health impacts.

## Author contributions

YiZ: Writing – original draft, Writing – review & editing. XZ: Conceptualization, Writing – original draft, Writing – review & editing. JZ: Writing – original draft, Writing – review & editing. YaZ: Conceptualization, Writing – review & editing. YW: Conceptualization, Writing – original draft, Writing – review & editing.

## References

[ref1] Abhishek A. Deb S. D. Jha R. K. Sinha R. Jha K. (2023). Classification of leukemia using fine tuned VGG16. In: 2023 International Conference on Signal Processing, Computation, Electronics, Power and Telecommunication (IConSCEPT): 1–5.

[ref2] Al-Aamri H. M. Irving H. R. Bradley C. Meehan-Andrews T. (2021). Intrinsic and extrinsic apoptosis responses in leukaemia cells following daunorubicin treatment. BMC Cancer 21:438. doi: 10.1186/s12885-021-08167-y, PMID: 33879127 PMC8059319

[ref3] Al-Aamri H. M. Ku H. Irving H. R. Tucci J. Meehan-Andrews T. Bradley C. (2019). Time dependent response of daunorubicin on cytotoxicity, cell cycle and DNA repair in acute lymphoblastic leukaemia. BMC Cancer 19:179. doi: 10.1186/s12885-019-5377-y, PMID: 30813936 PMC6391779

[ref4] Alexander T. C. Krull K. R. (2021). Effects of chemotherapy for acute lymphoblastic leukemia on cognitive function in animal models of contemporary protocols: a systematic literature review. Neurosci. Biobehav. Rev. 129, 206–217. doi: 10.1016/j.neubiorev.2021.07.033, PMID: 34352229 PMC8451158

[ref5] Alexander J. L. Wilson I. D. Teare J. Marchesi J. R. Nicholson J. K. Kinross J. M. (2017). Gut microbiota modulation of chemotherapy efficacy and toxicity. Nat. Rev. Gastroenterol. Hepatol. 14, 356–365. doi: 10.1038/nrgastro.2017.20 28270698

[ref6] Badgeley A. Anwar H. Modi K. Murphy P. Lakshmikuttyamma A. (2021). Effect of probiotics and gut microbiota on anti-cancer drugs: mechanistic perspectives. Biochim. Biophys. Acta Rev. Cancer 1875:188494. doi: 10.1016/j.bbcan.2020.188494, PMID: 33346129

[ref7] Banna G. L. Torino F. Marletta F. Santagati M. Salemi R. Cannarozzo E.. (2017). *Lactobacillus rhamnosus* GG: an overview to explore the rationale of its use in cancer. Front. Pharmacol. 8:603. doi: 10.3389/fphar.2017.00603, PMID: 28919861 PMC5585742

[ref8] Bates J. M. Akerlund J. Mittge E. Guillemin K. (2007). Intestinal alkaline phosphatase detoxifies lipopolysaccharide and prevents inflammation in zebrafish in response to the gut microbiota. Cell Host Microbe 2, 371–382. doi: 10.1016/j.chom.2007.10.010, PMID: 18078689 PMC2730374

[ref9] Bedhiafi T. Inchakalody V. P. Fernandes Q. Mestiri S. Billa N. Uddin S.. (2022). The potential role of vitamin c in empowering cancer immunotherapy. Biomed. Pharmacother. 146:112553. doi: 10.1016/j.biopha.2021.112553, PMID: 34923342

[ref10] Bertoli S. Picard M. Bérard E. Griessinger E. Larrue C. Mouchel P. L.. (2018). Dexamethasone in hyperleukocytic acute myeloid leukemia. Haematologica 103, 988–998. doi: 10.3324/haematol.2017.184267, PMID: 29519869 PMC6058767

[ref11] Beyar-Katz O. Gill S. (2018). Novel approaches to acute myeloid leukemia immunotherapy. Clin. Cancer Res. 24, 5502–5515. doi: 10.1158/1078-0432.Ccr-17-3016 29903894

[ref12] Bingula R. Filaire M. Radosevic-Robin N. Berthon J. Y. Bernalier-Donadille A. Vasson M. P.. (2018). Characterisation of gut, lung, and upper airways microbiota in patients with non-small cell lung carcinoma: study protocol for case-control observational trial. Medicine (Baltimore) 97:e13676. doi: 10.1097/md.0000000000013676, PMID: 30558074 PMC6320062

[ref13] Bispo J. A. B. Pinheiro P. S. Kobetz E. K. (2020). Epidemiology and etiology of leukemia and lymphoma. Cold Spring Harb. Perspect. Med. 10:a034819. doi: 10.1101/cshperspect.a034819, PMID: 31727680 PMC7263093

[ref14] Bosch F. Dalla-Favera R. (2019). Chronic lymphocytic leukaemia: from genetics to treatment. Nat. Rev. Clin. Oncol. 16, 684–701. doi: 10.1038/s41571-019-0239-8 31278397

[ref15] Canale F. P. Basso C. Antonini G. Perotti M. Li N. Sokolovska A.. (2021). Metabolic modulation of tumours with engineered bacteria for immunotherapy. Nature 598, 662–666. doi: 10.1038/s41586-021-04003-2 34616044

[ref16] Carnovali M. Valli R. Banfi G. Porta G. Mariotti M. (2021). Soybean meal-dependent intestinal inflammation induces different patterns of bone-loss in adult zebrafish scale. Biomedicines 9:393. doi: 10.3390/biomedicines9040393, PMID: 33917641 PMC8067592

[ref17] Caru M. Corbin D. Périé D. Lemay V. Delfrate J. Drouin S.. (2019). Doxorubicin treatments induce significant changes on the cardiac autonomic nervous system in childhood acute lymphoblastic leukemia long-term survivors. Clin. Res. Cardiol. 108, 1000–1008. doi: 10.1007/s00392-019-01427-9, PMID: 30778669

[ref18] Chang J. H. C. Poppe M. M. Hua C. H. Marcus K. J. Esiashvili N. (2021). Acute lymphoblastic leukemia. Pediatr. Blood Cancer 68:e28371. doi: 10.1002/pbc.28371 33818880

[ref19] Chen C. Zhang J. (2024). Enhancing leukemia treatment: the role of combined therapies based on amino acid starvation. Cancers 16:1171. doi: 10.3390/cancers16061171, PMID: 38539506 PMC10969718

[ref20] Choe H. Ferrara J. L. M. (2021). New therapeutic targets and biomarkers for acute graft-versus-host disease (gvhd). Expert Opin. Ther. Targets 25, 761–771. doi: 10.1080/14728222.2021.1992383, PMID: 34669521 PMC8602762

[ref21] Cholan P. M. Han A. Woodie B. R. Watchon M. Kurz A. R. Laird A. S.. (2020). Conserved anti-inflammatory effects and sensing of butyrate in zebrafish. Gut Microbes 12, 1824563–1824511. doi: 10.1080/19490976.2020.1824563, PMID: 33064972 PMC7575005

[ref22] Chougule R. A. Shah K. Moharram S. A. Vallon-Christersson J. Kazi J. U. (2019). Glucocorticoid-resistant B cell acute lymphoblastic leukemia displays receptor tyrosine kinase activation. NPJ Genom. Med. 4:7. doi: 10.1038/s41525-019-0082-y 30962949 PMC6449402

[ref23] Chrysostomou D. Roberts L. A. Marchesi J. R. Kinross J. M. (2023). Gut microbiota modulation of efficacy and toxicity of cancer chemotherapy and immunotherapy. Gastroenterology 164, 198–213. doi: 10.1053/j.gastro.2022.10.018 36309208

[ref24] Chuah L. O. Foo H. L. Loh T. C. Mohammed Alitheen N. B. Yeap S. K. Abdul Mutalib N. E.. (2019). Postbiotic metabolites produced by *lactobacillus plantarum* strains exert selective cytotoxicity effects on cancer cells. BMC Complement. Altern. Med. 19:114. doi: 10.1186/s12906-019-2528-2, PMID: 31159791 PMC6547513

[ref25] Ci T. Zhang W. Qiao Y. Li H. Zang J. Li H.. (2022). Delivery strategies in treatments of leukemia. Chem. Soc. Rev. 51, 2121–2144. doi: 10.1039/d1cs00755f 35188506

[ref26] Cong L. Ran F. A. Cox D. Lin S. Barretto R. Habib N.. (2013). Multiplex genome engineering using crispr/cas systems. Science 339, 819–823. doi: 10.1126/science.1231143, PMID: 23287718 PMC3795411

[ref27] Cong J. Zhu J. Zhang C. Li T. Liu K. Liu D.. (2019). Chemotherapy alters the phylogenetic molecular ecological networks of intestinal microbial communities. Front. Microbiol. 10:1008. doi: 10.3389/fmicb.2019.01008, PMID: 31134034 PMC6524687

[ref28] Conneely S. E. Cooper S. L. Rau R. E. (2020). Use of allopurinol to mitigate 6-mercaptopurine associated gastrointestinal toxicity in acute lymphoblastic leukemia. Front. Oncol. 10:1129. doi: 10.3389/fonc.2020.01129, PMID: 32766146 PMC7378397

[ref29] Daver N. Alotaibi A. S. Bücklein V. Subklewe M. (2021). T-cell-based immunotherapy of acute myeloid leukemia: current concepts and future developments. Leukemia 35, 1843–1863. doi: 10.1038/s41375-021-01253-x, PMID: 33953290 PMC8257483

[ref30] De Ruysscher D. Niedermann G. Burnet N. G. Siva S. Lee A. W. Hegi-Johnson F. (2019). Radiotherapy toxicity. Nat. Rev. Dis. Prim. 5:13. doi: 10.1038/s41572-019-0064-5 30792503

[ref31] Dhyani P. Quispe C. Sharma E. Bahukhandi A. Sati P. Attri D. C.. (2022). Anticancer potential of alkaloids: a key emphasis to colchicine, vinblastine, vincristine, vindesine, vinorelbine and vincamine. Cancer Cell Int. 22:206. doi: 10.1186/s12935-022-02624-9, PMID: 35655306 PMC9161525

[ref32] Di Francia R. Crisci S. De Monaco A. Cafiero C. Re A. Iaccarino G.. (2021). Response and toxicity to cytarabine therapy in leukemia and lymphoma: from dose puzzle to pharmacogenomic biomarkers. Cancers (Basel) 13:966. doi: 10.3390/cancers13050966, PMID: 33669053 PMC7956511

[ref33] Dicks L. M. T. Vermeulen W. (2022). Do bacteria provide an alternative to cancer reatment and what role does lactic acid bacteria play? Microorganisms 10:1733. doi: 10.3390/microorganisms10091733, PMID: 36144335 PMC9501580

[ref34] Dolatkhah R. Jam E. I. Nikanfar A. Esfahani A. Chavooshi S. H. Nejati B.. (2019). Outcome analysis of acute myeloid leukemia patients treated with high dose daunorubicin. Biomed. Res. Ther. 6, 3347–3351. doi: 10.15419/bmrat.v6i9.562

[ref35] Eghbali A. Ghaffari K. Khalilpour A. Afzal R. R. Eghbali A. Ghasemi A. (2023). The effects of lactocare synbiotic administration on chemotherapy-induced nausea, vomiting, diarrhea, and constipation in children with all: a double-blind randomized clinical trial. Pediatr. Blood Cancer 70:e30328. doi: 10.1002/pbc.30328, PMID: 36975174

[ref36] Garbacz K. (2022). Anticancer activity of lactic acid bacteria. Semin. Cancer Biol. 86, 356–366. doi: 10.1016/j.semcancer.2021.12.013, PMID: 34995799

[ref37] Gately S. (2019). Human microbiota and personalized cancer treatments: role of commensal microbes in treatment outcomes for cancer patients. Cancer Treat. Res. 178, 253–264. doi: 10.1007/978-3-030-16391-4_10, PMID: 31209849

[ref38] Gavillet M. Carr Klappert J. Spertini O. Blum S. (2020). Acute leukemia in the time of covid-19. Leuk. Res. 92:106353. doi: 10.1016/j.leukres.2020.106353, PMID: 32251934 PMC7138175

[ref40] Gibson R. J. Keefe D. M. Lalla R. V. Bateman E. Blijlevens N. Fijlstra M.. (2013). Systematic review of agents for the management of gastrointestinal mucositis in cancer patients. Support Care Cancer 21, 313–326. doi: 10.1007/s00520-012-1644-z 23142924

[ref41] Goldman J. M. Melo J. V. (2003). Chronic myeloid leukemia--advances in biology and new approaches to treatment. N. Engl. J. Med. 349, 1451–1464. doi: 10.1056/NEJMra020777 14534339

[ref42] Greathouse K. L. Wyatt M. Johnson A. J. Toy E. P. Khan J. M. Dunn K.. (2022). Diet-microbiome interactions in cancer treatment: opportunities and challenges for precision nutrition in cancer. Neoplasia 29:100800. doi: 10.1016/j.neo.2022.100800, PMID: 35500546 PMC9065883

[ref43] Gu Z. Li F. Liu Y. Jiang M. Zhang L. He L.. (2021). Exosome-like nanoparticles from *lactobacillus rhamnosus* GG protect against alcohol-associated liver disease through intestinal aryl hydrocarbon receptor in mice. Hepatol. Commun. 5, 846–864. doi: 10.1002/hep4.1679, PMID: 34027273 PMC8122379

[ref44] Haapaniemi E. Botla S. Persson J. Schmierer B. Taipale J. (2018). Crispr-cas9 genome editing induces a p53-mediated DNA damage response. Nat. Med. 24, 927–930. doi: 10.1038/s41591-018-0049-z, PMID: 29892067

[ref45] Hamada T. Zhang X. Mima K. Bullman S. Sukawa Y. Nowak J. A.. (2018). *Fusobacterium nucleatum* in colorectal cancer relates to immune response differentially by tumor microsatellite instability status. Cancer Immunol. Res. 6, 1327–1336. doi: 10.1158/2326-6066.Cir-18-0174, PMID: 30228205 PMC6215508

[ref46] Hamouda N. Sano T. Oikawa Y. Ozaki T. Shimakawa M. Matsumoto K.. (2017). Apoptosis, dysbiosis and expression of inflammatory cytokines are sequential events in the development of 5-fluorouracil-induced intestinal mucositis in mice. Basic Clin. Pharmacol. Toxicol. 121, 159–168. doi: 10.1111/bcpt.12793, PMID: 28374966

[ref47] Hansen B. A. Wendelbo Ø. Bruserud Ø. Hemsing A. L. Mosevoll K. A. Reikvam H. (2020). Febrile neutropenia in acute leukemia. Epidemiology, etiology, pathophysiology and treatment. Mediterr. J. Hematol. Infect. Dis. 12:e2020009. doi: 10.4084/mjhid.2020.009, PMID: 31934319 PMC6951355

[ref48] Harrison N. R. Laroche F. J. Gutierrez A. Feng H. (2016). Zebrafish models of human leukemia: technological advances and mechanistic insights. Adv. Exp. Med. Biol. 916, 335–369. doi: 10.1007/978-3-319-30654-4_15, PMID: 27165361 PMC4933302

[ref49] He X. Zhao S. Li Y. (2021). *Faecalibacterium prausnitzii*: A next-generation probiotic in gut disease improvement. Can. J. Infect. Dis. Med. Microbiol. 2021, 1–10. doi: 10.1155/2021/6666114

[ref50] Huang H. (2022). Research on the correlation between intestinal microecology and leukemia. Highlights Sci. Eng. Technol. 6, 339–347. doi: 10.54097/hset.v6i.979

[ref51] Huang S. He C. Li J. Gao Y. Z. Wang Z. Wei Y. (2023). Emerging paradigms in exploring the interactions among diet, probiotics, and cancer immunotherapeutic response. Innovation (Camb) 4:100456. doi: 10.1016/j.xinn.2023.100456, PMID: 37485077 PMC10362515

[ref52] Huang J. Huang X. Huang J. (2022). Car-t cell therapy for hematological malignancies: limitations and optimization strategies. Front. Immunol. 13:1019115. doi: 10.3389/fimmu.2022.1019115, PMID: 36248810 PMC9557333

[ref53] Huang Y. Yang W. Liu H. Duan J. Zhang Y. Liu M.. (2012). Effect of high-dose methotrexate chemotherapy on intestinal *Bifidobacteria*, *Lactobacillus* and *Escherichia coli* in children with acute lymphoblastic leukemia. Exp. Biol. Med. (Maywood) 237, 305–311. doi: 10.1258/ebm.2011.011297, PMID: 22362190

[ref54] Imaoka A. Shima T. Kato K. Mizuno S. Uehara T. Matsumoto S.. (2008). Anti-inflammatory activity of probiotic *bifidobacterium*: enhancement of IL-10 production in peripheral blood mononuclear cells from ulcerative colitis patients and inhibition of IL-8 secretion in HT-29 cells. World J. Gastroenterol. 14, 2511–2516. doi: 10.3748/wjg.14.2511, PMID: 18442197 PMC2708361

[ref55] Inaba H. Pui C. H. (2019). Immunotherapy in pediatric acute lymphoblastic leukemia. Cancer Metastasis Rev. 38, 595–610. doi: 10.1007/s10555-019-09834-0, PMID: 31811553 PMC6995750

[ref56] Iyer C. Kosters A. Sethi G. Kunnumakkara A. B. Aggarwal B. B. Versalovic J. (2008). Probiotic *Lactobacillus reuteri* promotes TNF-induced apoptosis in human myeloid leukemia-derived cells by modulation of NF-κB and MAPK signalling. Cell. Microbiol. 10, 1442–1452. doi: 10.1111/j.1462-5822.2008.01137.x 18331465

[ref57] Jaime-Pérez J. C. Ramos-Dávila E. M. Meléndez-Flores J. D. González-Treviño M. Gómez-Almaguer D. (2023). Assessing the efficacy of mitoxantrone and doxorubicin as frontline anthracyclines during induction therapy of newly diagnosed acute promyelocytic leukemia. Hematol. Oncol. Stem Cell Ther. 17, 13–20. doi: 10.56875/2589-0646.1090 37581460

[ref58] Javdan B. Lopez J. G. Chankhamjon P. Lee Y. J. Hull R. Wu Q.. (2020). Personalized mapping of drug metabolism by the human gut microbiome. Cell 181, 1661–1679.e22. doi: 10.1016/j.cell.2020.05.001, PMID: 32526207 PMC8591631

[ref59] Jaye K. Li C. G. Chang D. Bhuyan D. J. (2022). The role of key gut microbial metabolites in the development and treatment of cancer. Gut Microbes 14:2038865. doi: 10.1080/19490976.2022.2038865, PMID: 35220885 PMC8890435

[ref60] Jenq R. R. Taur Y. Devlin S. M. Ponce D. M. Goldberg J. D. Ahr K. F.. (2015). Intestinal blautia is associated with reduced death from graft-versus-host disease. Biol. Blood Marrow Transplant. 21, 1373–1383. doi: 10.1016/j.bbmt.2015.04.016 25977230 PMC4516127

[ref61] Jin Y.-B. Cao X. Shi C.-W. Feng B. Huang H.-B. Jiang Y.-L.. (2021). *Lactobacillus rhamnosus* GG promotes early B lineage development and IgA production in the lamina propria in piglets. J. Immunol. 207, 2179–2191. doi: 10.4049/jimmunol.2100102, PMID: 34497150

[ref62] Jin S. Xu J. Zou Y. Li X. Yu B. Han J.. (2023). Microbiome changes involves in mercaptopurine mediated anti-inflammatory response in acute lymphoblastic leukemia mice. Int. Immunopharmacol. 123:110782. doi: 10.1016/j.intimp.2023.110782, PMID: 37573688

[ref63] Juluri K. R. Siu C. Cassaday R. D. (2022). Asparaginase in the treatment of acute lymphoblastic leukemia in adults: current evidence and place in therapy. Blood Lymphat. Cancer 12, 55–79. doi: 10.2147/blctt.S342052, PMID: 35669980 PMC9166408

[ref64] Jurcic J. G. (2020). Targeted alpha-particle therapy for hematologic malignancies. Semin. Nucl. Med. 50, 152–161. doi: 10.1053/j.semnuclmed.2019.09.002 32172800

[ref65] Kaźmierczak-Siedlecka K. Roviello G. Catalano M. Polom K. (2021). Gut microbiota modulation in the context of immune-related aspects of *lactobacillus* spp. and *bifidobacterium* spp. in gastrointestinal cancers. Nutrients 13:2674. doi: 10.3390/nu13082674, PMID: 34444834 PMC8401094

[ref66] Lalic H. Aurer I. Batinic D. Visnjic D. Smoljo T. Babic A. (2022). Bendamustine: a review of pharmacology, clinical use and immunological effects (review). Oncol. Rep. 47:114. doi: 10.3892/or.2022.8325, PMID: 35506458 PMC9100486

[ref67] Laukkanen S. Veloso A. Yan C. Oksa L. Alpert E. J. Do D.. (2022). Therapeutic targeting of lck tyrosine kinase and mtor signaling in T-cell acute lymphoblastic leukemia. Blood 140, 1891–1906. doi: 10.1182/blood.2021015106, PMID: 35544598 PMC10082361

[ref68] Li Y. Wang J. Wang M. Gao Y. Jin C.-Y. Shi X.. (2021). Microbial profiling identifies potential key drivers in gastric cancer patients. Biotechnol. Biotechnol. Equip. 35, 496–503. doi: 10.1080/13102818.2021.1896384

[ref69] Li F. Wang H. Ye T. Guo P. Lin X. Hu Y.. (2024). Recent advances in material technology for leukaemia treatments. Adv. Mater.:2313955. doi: 10.1002/adma.202313955 38547845

[ref70] Liao Y. Wu Y. X. Tang M. Chen Y. W. Xie J. R. Du Y.. (2024). Microbes translocation from oral cavity to nasopharyngeal carcinoma in patients. Nat. Commun. 15:1645. doi: 10.1038/s41467-024-45518-2, PMID: 38388556 PMC10883945

[ref71] Lin Y.-W. Aplan P. D. (2004). Leukemic transformation. Cancer Biol. Ther. 3, 13–20. doi: 10.4161/cbt.3.1.537 14726677

[ref72] Liu Y. Li X. Yang Y. Liu Y. Wang S. Ji B.. (2021). Exploring gut microbiota in patients with colorectal disease based on 16s rRNA gene amplicon and shallow metagenomic sequencing. Front. Mol. Biosci. 8:703638. doi: 10.3389/fmolb.2021.703638, PMID: 34307461 PMC8299945

[ref73] Liu J. Wei Y. Jia W. Can C. Wang R. Yang X.. (2022). Chenodeoxycholic acid suppresses aml progression through promoting lipid peroxidation via ROS/p38 MAPK/DGAT1 pathway and inhibiting M2 macrophage polarization. Redox Biol. 56:102452. doi: 10.1016/j.redox.2022.102452, PMID: 36084349 PMC9465103

[ref74] Liu W. Yi J. M. Liu Y. Chen C. Zhang K. X. Zhou C.. (2020). CDK6 is a potential prognostic biomarker in acute myeloid leukemia. Front. Genet. 11:600227. doi: 10.3389/fgene.2020.600227, PMID: 33597968 PMC7882723

[ref75] López-Gómez L. Alcorta A. Abalo R. (2023). Probiotics and probiotic-like agents against chemotherapy-induced intestinal mucositis: a narrative review. J. Pers. Med. 13:1487. doi: 10.3390/jpm13101487, PMID: 37888098 PMC10607965

[ref76] Lu K. Dong S. Wu X. Jin R. Chen H. (2021). Probiotics in cancer. Front. Oncol. 11:638148. doi: 10.3389/fonc.2021.638148, PMID: 33791223 PMC8006328

[ref77] Lu D. Yan J. Liu F. Ding P. Chen B. Lu Y.. (2019). Probiotics in preventing and treating chemotherapy-induced diarrhea: a meta-analysis. Asia Pac. J. Clin. Nutr. 28, 701–710. doi: 10.6133/apjcn.201912_28(4).0005, PMID: 31826366

[ref78] Ma T. Chen Y. Li L. J. Zhang L. S. (2021). Opportunities and challenges for gut microbiota in acute leukemia. Front. Oncol. 11:692951. doi: 10.3389/fonc.2021.692951, PMID: 34307157 PMC8293295

[ref79] Mahbub A. A. Maitre C. L. L. Haywood-Small S. Cross N. A. Jordan-Mahy N. (2019). Polyphenols enhance the activity of alkylating agents in leukaemia cell lines. Oncotarget 10, 4570–4586. doi: 10.18632/oncotarget.27068, PMID: 31360305 PMC6642044

[ref80] Malard F. Vekhoff A. Lapusan S. Isnard F. D'Incan-Corda E. Rey J.. (2021). Gut microbiota diversity after autologous fecal microbiota transfer in acute myeloid leukemia patients. Nat. Commun. 12:3084. doi: 10.1038/s41467-021-23376-6, PMID: 34035290 PMC8149453

[ref81] Mandelbaum N. Zhang L. Carasso S. Ziv T. Lifshiz-Simon S. Davidovich I.. (2023). Extracellular vesicles of the gram-positive gut symbiont *Bifidobacterium longum* induce immune-modulatory, anti-inflammatory effects. NPJ Biofilms Microbiomes 9:30. doi: 10.1038/s41522-023-00400-9, PMID: 37270554 PMC10239484

[ref82] Martyniak A. Zakrzewska Z. Schab M. Zawartka A. Wędrychowicz A. Skoczeń S.. (2023). Prevention and health benefits of prebiotics, probiotics and postbiotics in acute lymphoblastic leukemia. Microorganisms 11:1775. doi: 10.3390/microorganisms11071775, PMID: 37512947 PMC10384688

[ref83] McNeer N. A. Philip J. Geiger H. Ries R. E. Lavallée V. P. Walsh M.. (2019). Genetic mechanisms of primary chemotherapy resistance in pediatric acute myeloid leukemia. Leukemia 33, 1934–1943. doi: 10.1038/s41375-019-0402-3, PMID: 30760869 PMC6687545

[ref85] Michaelidesová A. Konířová J. Bartůněk P. Zíková M. (2019). Effects of radiation therapy on neural stem cells. Genes (Basel) 10:640. doi: 10.3390/genes10090640, PMID: 31450566 PMC6770913

[ref86] Mu X. Chen C. Dong L. Kang Z. Sun Z. Chen X.. (2023). Immunotherapy in leukaemia. Acta Biochim. Biophys. Sin. Shanghai 55, 974–987. doi: 10.3724/abbs.2023101, PMID: 37272727 PMC10326417

[ref87] Naci D. Berrazouane S. Barabé F. Aoudjit F. (2019). Cell adhesion to collagen promotes leukemia resistance to doxorubicin by reducing DNA damage through the inhibition of rac1 activation. Sci. Rep. 9:19455. doi: 10.1038/s41598-019-55934-w, PMID: 31857649 PMC6923425

[ref88] Naeem H. Hassan H. U. Shahbaz M. Imran M. Memon A. G. Hasnain A.. (2024). Role of probiotics against human cancers, inflammatory diseases, and other complex malignancies. J. Food Biochem. 2024, 1–23. doi: 10.1155/2024/6632209

[ref89] Nakkarach A. Foo H. L. Song A. A. Mutalib N. E. A. Nitisinprasert S. Withayagiat U. (2021). Anti-cancer and anti-inflammatory effects elicited by short chain fatty acids produced by *escherichia coli* isolated from healthy human gut microbiota. Microb. Cell Factories 20:36. doi: 10.1186/s12934-020-01477-z, PMID: 33546705 PMC7863513

[ref90] Ness K. K. Armenian S. H. Kadan-Lottick N. Gurney J. G. (2011). Adverse effects of treatment in childhood acute lymphoblastic leukemia: general overview and implications for long-term cardiac health. Expert Rev. Hematol. 4, 185–197. doi: 10.1586/ehm.11.8, PMID: 21495928 PMC3125981

[ref91] Nowak A. Paliwoda A. Błasiak J. (2019). Anti-proliferative, pro-apoptotic and anti-oxidative activity of *Lactobacillus* and *Bifidobacterium strains*: a review of mechanisms and therapeutic perspectives. Crit. Rev. Food Sci. Nutr. 59, 3456–3467. doi: 10.1080/10408398.2018.1494539, PMID: 30010390

[ref92] Olazagoitia-Garmendia A. Rojas-Márquez H. Sebastian-delaCruz M. Agirre-Lizaso A. Ochoa A. Mendoza-Gomez L. M.. (2024). M^6^ a methylated long noncoding RNA *LOC339803* regulates intestinal inflammatory response. Adv. Sci. (Weinh) 11:e2307928. doi: 10.1002/advs.202307928, PMID: 38273714 PMC10987157

[ref93] Org E. Parks B. W. Joo J. W. Emert B. Schwartzman W. Kang E. Y.. (2015). Genetic and environmental control of host-gut microbiota interactions. Genome Res. 25, 1558–1569. doi: 10.1101/gr.194118.115, PMID: 26260972 PMC4579341

[ref94] Page D. M. Wittamer V. Bertrand J. Y. Lewis K. L. Pratt D. N. Delgado N.. (2013). An evolutionarily conserved program of b-cell development and activation in zebrafish. Blood 122, e1–e11. doi: 10.1182/blood-2012-12-471029, PMID: 23861249 PMC3750348

[ref95] Przespolewski A. Szeles A. Wang E. S. (2018). Advances in immunotherapy for acute myeloid leukemia. Future Oncol. 14, 963–978. doi: 10.2217/fon-2017-0459 29542352

[ref97] Ren Z. Hong Y. Huo Y. Peng L. Lv H. Chen J.. (2022). Prospects of probiotic adjuvant drugs in clinical treatment. Nutrients 14:4723. doi: 10.3390/nu14224723, PMID: 36432410 PMC9697729

[ref98] Rensen N. Steur L. M. H. Grootenhuis M. A. van Eijkelenburg N. K. A. van der Sluis I. M. Dors N.. (2020). Parental functioning during maintenance treatment for childhood acute lymphoblastic leukemia: effects of treatment intensity and dexamethasone pulses. Pediatr. Blood Cancer 67:e28697. doi: 10.1002/pbc.28697, PMID: 32909677

[ref99] Reyna-Figueroa J. Barrón-Calvillo E. García-Parra C. Galindo-Delgado P. Contreras-Ochoa C. Lagunas-Martínez A.. (2019). Probiotic supplementation decreases chemotherapy-induced gastrointestinal side effects in patients with acute leukemia. J. Pediatr. Hematol. Oncol. 41, 468–472. doi: 10.1097/mph.0000000000001497, PMID: 31033786

[ref100] Ribatti D. d’Amati A. (2023). Hematopoiesis and mast cell development. Int. J. Mol. Sci. 24:10679. doi: 10.3390/ijms241310679, PMID: 37445862 PMC10342148

[ref101] Ridges S. Heaton W. L. Joshi D. Choi H. Eiring A. Batchelor L.. (2012). Zebrafish screen identifies novel compound with selective toxicity against leukemia. Blood 119, 5621–5631. doi: 10.1182/blood-2011-12-398818, PMID: 22490804 PMC3382926

[ref102] Robertson A. L. Avagyan S. Gansner J. M. Zon L. I. (2016). Understanding the regulation of vertebrate hematopoiesis and blood disorders - big lessons from a small fish. FEBS Lett. 590, 4016–4033. doi: 10.1002/1873-3468.12415, PMID: 27616157 PMC5483340

[ref103] Rodriguez-Arrastia M. Martinez-Ortigosa A. Rueda-Ruzafa L. Folch Ayora A. Ropero-Padilla C. (2021). Probiotic supplements on oncology patients' treatment-related side effects: a systematic review of randomized controlled trials. Int. J. Environ. Res. Public Health 18:4265. doi: 10.3390/ijerph18084265, PMID: 33920572 PMC8074215

[ref104] Routy B. Le Chatelier E. Derosa L. Duong C. P. M. Alou M. T. Daillère R.. (2018). Gut microbiome influences efficacy of PD-1-based immunotherapy against epithelial tumors. Science 359, 91–97. doi: 10.1126/science.aan3706, PMID: 29097494

[ref105] Sági V. Makra N. Csoszánszki N. Decmann A. Szabó D. Garami M. (2022). The influence of the gut microbiome in paediatric cancer origin and treatment. Antibiotics (Basel) 11:1521. doi: 10.3390/antibiotics11111521, PMID: 36358176 PMC9686478

[ref106] Salzer W. Bostrom B. Messinger Y. Perissinotti A. J. Marini B. (2018). Asparaginase activity levels and monitoring in patients with acute lymphoblastic leukemia. Leuk. Lymphoma 59, 1797–1806. doi: 10.1080/10428194.2017.1386305, PMID: 29045165

[ref107] Sánchez B. Delgado S. Blanco-Míguez A. Lourenço A. Gueimonde M. Margolles A. (2017). Probiotics, gut microbiota, and their influence on host health and disease. Mol. Nutr. Food Res. 61:1600240. doi: 10.1002/mnfr.201600240 27500859

[ref108] Sarkar A. McInroy C. J. A. Harty S. Raulo A. Ibata N. G. O. Valles-Colomer M.. (2024). Microbial transmission in the social microbiome and host health and disease. Cell 187, 17–43. doi: 10.1016/j.cell.2023.12.014, PMID: 38181740 PMC10958648

[ref109] Sharma V. Sharma N. Sheikh I. Kumar V. Sehrawat N. Yadav M.. (2021). Probiotics and prebiotics having broad spectrum anticancer therapeutic potential: recent trends and future perspectives. Curr. Pharmacol. Rep. 7, 67–79. doi: 10.1007/s40495-021-00252-x

[ref110] Shi M. Su R. J. Parmar K. P. Chaudhry R. Sun K. Rao J.. (2019). Cd123: a novel biomarker for diagnosis and treatment of leukemia. Cardiovasc. Hematol. Disord. Drug Targets 19, 195–204. doi: 10.2174/1871529x19666190627100613, PMID: 31244444

[ref111] Silva R. A. M. de Mendonça R. M. H. Dos Santos Aguiar S. Yajima J. C. Marson F. A. L. Brandalise S. R.. (2022). Induction therapy for acute lymphoblastic leukemia: incidence and risk factors for bloodstream infections. Support Care Cancer 30, 695–702. doi: 10.1007/s00520-021-06471-8, PMID: 34363492

[ref112] Singh A. Alexander S. G. Martin S. (2023). Gut microbiome homeostasis and the future of probiotics in cancer immunotherapy. Front. Immunol. 14:1114499. doi: 10.3389/fimmu.2023.1114499, PMID: 37261348 PMC10228691

[ref113] Sivan A. Corrales L. Hubert N. Williams J. B. Aquino-Michaels K. Earley Z. M.. (2015). Commensal bifidobacterium promotes antitumor immunity and facilitates anti-PD-L1 efficacy. Science 350, 1084–1089. doi: 10.1126/science.aac4255 26541606 PMC4873287

[ref114] Śliżewska K. Markowiak-Kopeć P. Śliżewska W. (2020). The role of probiotics in cancer prevention. Cancers (Basel) 13:20. doi: 10.3390/cancers13010020, PMID: 33374549 PMC7793079

[ref115] Song Y. Himmel B. Öhrmalm L. Gyarmati P. (2020). The microbiota in hematologic malignancies. Curr. Treat. Options in Oncol. 21:2. doi: 10.1007/s11864-019-0693-7 31927673

[ref116] Song Y. Perlman K. Gyarmati P. (2022). Microbial and host factors contribute to bloodstream infection in a pediatric acute lymphocytic leukemia mouse model. Heliyon 8:e11340. doi: 10.1016/j.heliyon.2022.e11340 36345525 PMC9636473

[ref117] Spencer C. N. McQuade J. L. Gopalakrishnan V. McCulloch J. A. Vetizou M. Cogdill A. P.. (2021). Dietary fiber and probiotics influence the gut microbiome and melanoma immunotherapy response. Science 374, 1632–1640. doi: 10.1126/science.aaz7015, PMID: 34941392 PMC8970537

[ref118] Suresh S. A. Ethiraj S. Rajnish K. N. (2020). Toxicity analysis of recombinant L-asparaginase I and II in zebrafish. Indian J. Microbiol. 60, 535–538. doi: 10.1007/s12088-020-00890-7, PMID: 33088004 PMC7539244

[ref119] Swimm A. Giver C. R. DeFilipp Z. Rangaraju S. Sharma A. Ulezko Antonova A.. (2018). Indoles derived from intestinal microbiota act via type I interferon signaling to limit graft-versus-host disease. Blood 132, 2506–2519. doi: 10.1182/blood-2018-03-838193, PMID: 30257880 PMC6284212

[ref120] Tan S. Li D. Zhu X. (2020). Cancer immunotherapy: pros, cons and beyond. Biomed. Pharmacother. 124:109821. doi: 10.1016/j.biopha.2020.109821, PMID: 31962285

[ref121] Tan J. Zhao L. Wang G. Li T. Li D. Xu Q.. (2018). Human MLL-AF9 overexpression induces aberrant hematopoietic expansion in zebrafish. Biomed. Res. Int. 2018:6705842. doi: 10.1155/2018/6705842, PMID: 30003105 PMC5998191

[ref122] Tanguay R. L. (2018). The rise of zebrafish as a model for toxicology. Toxicol. Sci. 163, 3–4. doi: 10.1093/toxsci/kfx295 29718442

[ref123] Tebbi C. K. (2021). Etiology of acute leukemia: a review. Cancers (Basel) 13:2256. doi: 10.3390/cancers13092256, PMID: 34066700 PMC8125807

[ref124] Teuffel O. Kuster S. P. Hunger S. P. Conter V. Hitzler J. Ethier M. C.. (2011). Dexamethasone versus prednisone for induction therapy in childhood acute lymphoblastic leukemia: a systematic review and meta-analysis. Leukemia 25, 1232–1238. doi: 10.1038/leu.2011.84, PMID: 21527934

[ref125] Trujillo E. B. Dixon S. W. Claghorn K. Levin R. M. Mills J. B. Spees C. K. (2018). Closing the gap in nutrition care at outpatient cancer centers: ongoing initiatives of the oncology nutrition dietetic practice group. J. Acad. Nutr. Diet. 118, 749–760. doi: 10.1016/j.jand.2018.02.010, PMID: 29576094

[ref126] Tuo Y. Jiang S. Qian F. Mu G. Liu P. Guo Y.. (2015). Short communication: Antiproliferative effect of 8 different lactobacillus strains on K562 cells. J. Dairy Sci. 98, 106–110. doi: 10.3168/jds.2014-8767, PMID: 25465570

[ref127] Uesugi T. Mori S. Miyanaga K. Yamamoto N. (2023). Groel secreted from *bacillus subtilis* natto exerted a crucial role for anti-inflammatory IL-10 induction in THP-1 cells. Microorganisms 11:1281. doi: 10.3390/microorganisms11051281, PMID: 37317255 PMC10221570

[ref128] Uribe-Herranz M. Klein-González N. Rodríguez-Lobato L. G. Juan M. de Larrea C. F. (2021). Gut microbiota influence in hematological malignancies: from genesis to cure. Int. J. Mol. Sci. 22:1026. doi: 10.3390/ijms22031026, PMID: 33498529 PMC7864170

[ref129] van Nood E. Vrieze A. Nieuwdorp M. Fuentes S. Zoetendal E. G. de Vos W. M.. (2013). Duodenal infusion of donor feces for recurrent *clostridium difficile*. N. Engl. J. Med. 368, 407–415. doi: 10.1056/NEJMoa1205037 23323867

[ref130] Viaud S. Saccheri F. Mignot G. Yamazaki T. Daillère R. Hannani D.. (2013). The intestinal microbiota modulates the anticancer immune effects of cyclophosphamide. Science 342, 971–976. doi: 10.1126/science.1240537, PMID: 24264990 PMC4048947

[ref131] Vincent R. L. Gurbatri C. R. Li F. Vardoshvili A. Coker C. Im J.. (2023). Probiotic-guided car-t cells for solid tumor targeting. Science 382, 211–218. doi: 10.1126/science.add7034, PMID: 37824640 PMC10915968

[ref132] Vivarelli S. Salemi R. Candido S. Falzone L. Santagati M. Stefani S.. (2019). Gut microbiota and cancer: from pathogenesis to therapy. Cancers (Basel) 11:38. doi: 10.3390/cancers11010038, PMID: 30609850 PMC6356461

[ref133] Wang F. Lv H. Zhao B. Zhou L. Wang S. Luo J.. (2019). Iron and leukemia: new insights for future treatments. J. Exp. Clin. Cancer Res. 38:406. doi: 10.1186/s13046-019-1397-3, PMID: 31519186 PMC6743129

[ref134] Wang R. Yang X. Liu J. Zhong F. Zhang C. Chen Y.. (2022). Gut microbiota regulates acute myeloid leukaemia via alteration of intestinal barrier function mediated by butyrate. Nat. Commun. 13:2522. doi: 10.1038/s41467-022-30240-8 35534496 PMC9085760

[ref135] Wei Y. Li X. Ji B. Qu L. (2022). Recent advances on the recovery, modulation and synthetic biology of gut microbiota and hosts. Sci Sin Vitae 52, 249–265. doi: 10.1360/SSV-2021-0088

[ref136] Wen Y. Jin R. Chen H. (2019). Interactions between gut microbiota and acute childhood leukemia. Front. Microbiol. 10:1300. doi: 10.3389/fmicb.2019.01300, PMID: 31275258 PMC6593047

[ref137] Wieërs G. Belkhir L. Enaud R. Leclercq S. Philippart de Foy J. M. Dequenne I.. (2019). How probiotics affect the microbiota. Front. Cell. Infect. Microbiol. 9:454. doi: 10.3389/fcimb.2019.00454, PMID: 32010640 PMC6974441

[ref138] Wollowski I. Rechkemmer G. Pool-Zobel B. L. (2001). Protective role of probiotics and prebiotics in colon cancer. Am. J. Clin. Nutr. 73, 451s–455s. doi: 10.1093/ajcn/73.2.451s, PMID: 11157356

[ref139] Xia C. Su J. Liu C. Mai Z. Yin S. Yang C.. (2023). Human microbiomes in cancer development and therapy. MedComm (2020) 4:e221. doi: 10.1002/mco2.221, PMID: 36860568 PMC9969057

[ref141] Yang Q. Y. Hu Y. H. Guo H. L. Xia Y. Zhang Y. Fang W. R.. (2021). Vincristine-induced peripheral neuropathy in childhood acute lymphoblastic leukemia: genetic variation as a potential risk factor. Front. Pharmacol. 12:771487. doi: 10.3389/fphar.2021.771487, PMID: 34955843 PMC8696478

[ref142] Yang Y. Li X. Yang Y. Shoaie S. Zhang C. Ji B.. (2021). Advances in the relationships between cow's milk protein allergy and gut microbiota in infants. Front. Microbiol. 12:716667. doi: 10.3389/fmicb.2021.716667, PMID: 34484158 PMC8415629

[ref143] Ye L. Mueller O. Bagwell J. Bagnat M. Liddle R. A. Rawls J. F. (2019). High fat diet induces microbiota-dependent silencing of enteroendocrine cells. eLife 8:e48479. doi: 10.7554/eLife.48479, PMID: 31793875 PMC6937151

[ref144] Yi Z. N. Chen X. K. Ma A. C. (2022). Modeling leukemia with zebrafish (danio rerio): towards precision medicine. Exp. Cell Res. 421:113401. doi: 10.1016/j.yexcr.2022.113401, PMID: 36306826

[ref145] Yin T. Zhang X. Iwatani S. Miyanaga K. Yamamoto N. (2023). Uptake of levilactobacillus brevis jcm 1059 by THP-1 cells via interaction between SLpB and CAP-1 promotes cytokine production. Microorganisms 11:247. doi: 10.3390/microorganisms11020247, PMID: 36838212 PMC9962577

[ref146] Yu D. Yu X. Ye A. Xu C. Li X. Geng W.. (2021). Profiling of gut microbial dysbiosis in adults with myeloid leukemia. FEBS Open Bio 11, 2050–2059. doi: 10.1002/2211-5463.13193, PMID: 33993646 PMC8406483

[ref147] Yuan Y. Yang Y. Xiao L. Qu L. Zhang X. Wei Y. (2023). Advancing insights into probiotics during vegetable fermentation. Food Secur. 12:3789. doi: 10.3390/foods12203789, PMID: 37893682 PMC10606808

[ref148] Yue B. Gao R. Wang Z. Dou W. (2021). Microbiota-host-irinotecan axis: a new insight toward irinotecan chemotherapy. Front. Cell. Infect. Microbiol. 11:710945. doi: 10.3389/fcimb.2021.710945, PMID: 34722328 PMC8553258

[ref149] Zhang X. Miao Q. Pan C. Yin J. Wang L.-L. Qu L.. (2023). Research advances in probiotic fermentation of chinese herbal medicines. iMeta 2:e93. doi: 10.1002/imt2.93, PMID: 38868438 PMC10989925

[ref150] Zhang C. H. Qi H. Li B. Zhang S. Y. Zhang L. Q. Wang J. D. (2022). Two new 22-membered macrolides from streptomyces sp. Hu210. J. Antibiot. (Tokyo) 75, 650–653. doi: 10.1038/s41429-022-00563-1, PMID: 36109668

[ref151] Zhong X. Li J. Lu F. Zhang J. Guo L. (2022). Application of zebrafish in the study of the gut microbiome. Animal Model Exp. Med. 5, 323–336. doi: 10.1002/ame2.12227, PMID: 35415967 PMC9434591

[ref152] Zhou D. Xu W. Ma H. Zhao C. Hu Y. Zhao Y.. (2022). Bendamustine versus chlorambucil in treatment of chronic lymphocytic leukaemia in China: a randomized, open-label, parallel-controlled, phase III clinical trial. Investig. New Drugs 40, 349–360. doi: 10.1007/s10637-021-01206-2, PMID: 35031896

[ref153] Zhou Y. Zhou C. Zhang A. (2022). Gut microbiota in acute leukemia: current evidence and future directions. Front. Microbiol. 13:1045497. doi: 10.3389/fmicb.2022.1045497, PMID: 36532458 PMC9751036

